# Impaired neural replay of inferred relationships in schizophrenia

**DOI:** 10.1016/j.cell.2021.06.012

**Published:** 2021-08-05

**Authors:** Matthew M. Nour, Yunzhe Liu, Atheeshaan Arumuham, Zeb Kurth-Nelson, Raymond J. Dolan

**Affiliations:** 1 Max Planck University College London Centre for Computational Psychiatry and Ageing Research, London WC1B 5EH, UK; 2 Wellcome Centre for Human Neuroimaging (WCHN), University College London, London WC1N 3AR, UK; 3 Department of Psychosis Studies, Institute of Psychiatry Psychology and Neuroscience, King’s College London, London SE5 8AF, UK; 4 State Key Laboratory of Cognitive Neuroscience and Learning, IDG/McGovern Institute for Brain Research, Beijing Normal University, Beijing 100875, China; 5 Chinese Institute for Brain Research, Beijing 102206, China; 6 Deepmind, London NC1 4AG, UK; 7 BIH Visiting Professor, Stiftung Charité, Department of Psychiatry and Psychotherapy, Campus Charité Mitte, Charité – Universitätsmedizin, Berlin, Germany

**Keywords:** schema, model-based inference, mental simulation, cognitive map, psychosis

## Abstract

An ability to build structured mental maps of the world underpins our capacity to imagine relationships between objects that extend beyond experience. In rodents, such representations are supported by sequential place cell reactivations during rest, known as replay. Schizophrenia is proposed to reflect a compromise in structured mental representations, with animal models reporting abnormalities in hippocampal replay and associated ripple activity during rest. Here, utilizing magnetoencephalography (MEG), we tasked patients with schizophrenia and control participants to infer unobserved relationships between objects by reorganizing visual experiences containing these objects. During a post-task rest session, controls exhibited fast spontaneous neural reactivation of presented objects that replayed inferred relationships. Replay was coincident with increased ripple power in hippocampus. Patients showed both reduced replay and augmented ripple power relative to controls, convergent with findings in animal models. These abnormalities are linked to impairments in behavioral acquisition and subsequent neural representation of task structure.

## Highlights

• Patients with schizophrenia show impairments in inferring sequential relationships
• Patients show reduced neural replay, during rest, for inferred structure
• Replay-associated ripple power is elevated in patients, as in animal models
• Patients show blunted representations of task structure, related to ripple power

## Graphical abstract

**Figure:**
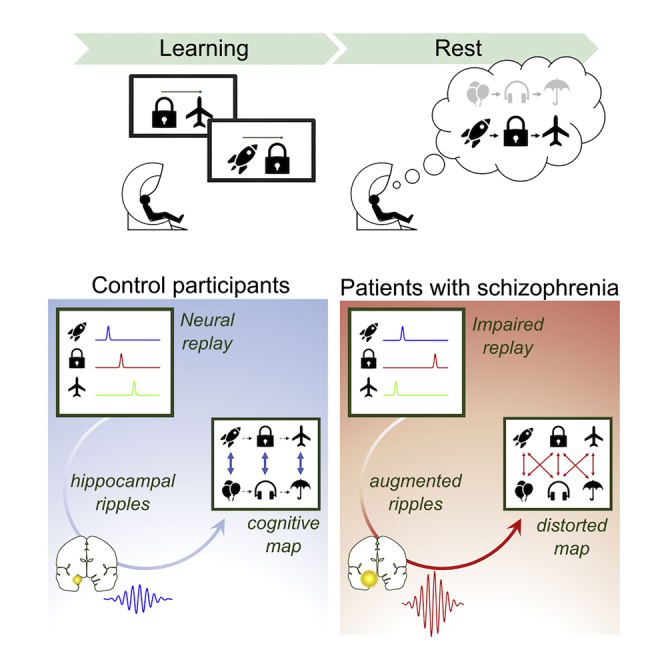

## Teaser

In support of schizophrenia reflecting compromised cognitive maps of the environment, patients with schizophrenia display abnormalities in hippocampal replay relative to controls.

## Introduction

In humans and other animals, “cognitive maps” (Craik, 1943; Tolman, 1948)—rich internal models of the world that account for the relationships between objects and events—are supported by neural representations within hippocampal-entorhinal cortex (HEC) (Behrens et al., 2018; Bellmund et al., 2018; O’Keefe and Nadel, 1978). This has been described best in rodents during active navigation, where subpopulations of HEC pyramidal neurons exhibit spatially localized firing fields (e.g., place and grid cells; Fyhn et al., 2004; Hafting et al., 2005; O’Keefe and Dostrovsky, 1971; Wilson and McNaughton, 1993). During subsequent rest periods a rapid sequential place cell reactivation (replay) recapitulates previously navigated trajectories, in addition to exploring novel paths that go beyond previous experience (Diba and Buzsáki, 2007; Foster, 2017; Foster and Wilson, 2006; Ólafsdóttir et al., 2015; Gupta et al., 2010; Lee and Wilson, 2002; Nádasdy et al., 1999; Wilson and McNaughton, 1994). In humans, functional MRI studies also reveal map-like signatures in HEC for non-spatial domains (Constantinescu et al., 2016; Garvert et al., 2017) and temporally structured spontaneous memory reactivations during rest (Schuck and Niv, 2019), suggesting analogous representations in conceptual and cognitive spaces (Whittington et al., 2020).

Recent advances now enable detection of spontaneous neural replay in humans noninvasively, using magnetoencephalography (MEG) (Kurth-Nelson et al., 2016; Liu et al., 2021a, 2019; Wimmer et al., 2020). This allows researchers to pose new questions relating to abstract and non-spatial forms of cognition in humans, which are difficult to address in rodents, in addition to pursuing pressing questions related to neural processes underlying neuropsychiatric conditions.

Here, we focus on the investigation of replay in schizophrenia, a debilitating neuropsychiatric disorder with lifetime prevalence approaching 1% (McCutcheon et al., 2020b). Distorted associative representations are proposed to underlie its clinical manifestations, including bizarre delusions and conceptual disorganization (Bleuler, 1911; McKenna, 2007). Affected patients show impairments in inferring unobserved relationships between task states (Adams et al., 2020; Titone et al., 2004) and using such knowledge for decision making (Morris et al., 2018). Moreover, these patients exhibit well-replicated structural (Brugger and Howes, 2017) and functional (Grace, 2016; McHugo et al., 2019; Schobel et al., 2013) hippocampal abnormalities. Of particular relevance are recent genetic mouse models of schizophrenia, which highlight abnormalities in hippocampal place cell reactivation and associated sharp wave ripple (SWR) complexes, during rest (Altimus et al., 2015; Suh et al., 2013; Zaremba et al., 2017).

Replay and SWRs contribute to consolidation of a cognitive map (Fernández-Ruiz et al., 2019; Joo and Frank, 2018; Roux et al., 2017; Schapiro et al., 2018) and might “stitch together” associative memories to enable reasoning about relationships that go beyond direct experience (Barron et al., 2020; Foster, 2017; Gupta et al., 2010; Liu et al., 2019). This raises an intriguing hypothesis that impaired replay in schizophrenia, and resulting neural map distortions, could provide a crucial conceptual link between neurobiological abnormalities and cognitive impairments involving sophisticated model-based reasoning (Adams et al., 2020; Titone et al., 2004). Inferences of this nature are intimately related to symptom manifestations such as paranoia and are not readily assessed by tasks where patients learn from direct experience alone (Maia and Frank, 2017), a situation where there is no requirement for an internal associative model (map) of the world.

We test a hypothesis of abnormal replay and cognitive map construction in schizophrenia, leveraging methodological advances that enable both detection of fast spontaneous neural replay (Kurth-Nelson et al., 2016; Liu et al., 2021a, 2019) and the representational content of neural responses (Barron et al., 2020; Deuker et al., 2016; Diedrichsen and Kriegeskorte, 2017; Luyckx et al., 2019) in humans. Using a non-spatial structure-learning task (Liu et al., 2019) and neural data derived from MEG, we measured spontaneous replay of inferred task structure during a post-learning awake rest period. We show that patients exhibit abnormalities in the temporal dynamics of spontaneous memory reactivations, and associated high-frequency (ripple-band) oscillations, during post-learning rest, mirroring those seen in animal models (Altimus et al., 2015; Suh et al., 2013; Zaremba et al., 2017). Importantly, these abnormalities relate to behavioral impairments specific to inferences about sequential relationships, as well as an impoverished neural representation of task structure after learning.

## Results

### Inferring structural relationships by applying an abstracted task template

**Figure 1:**
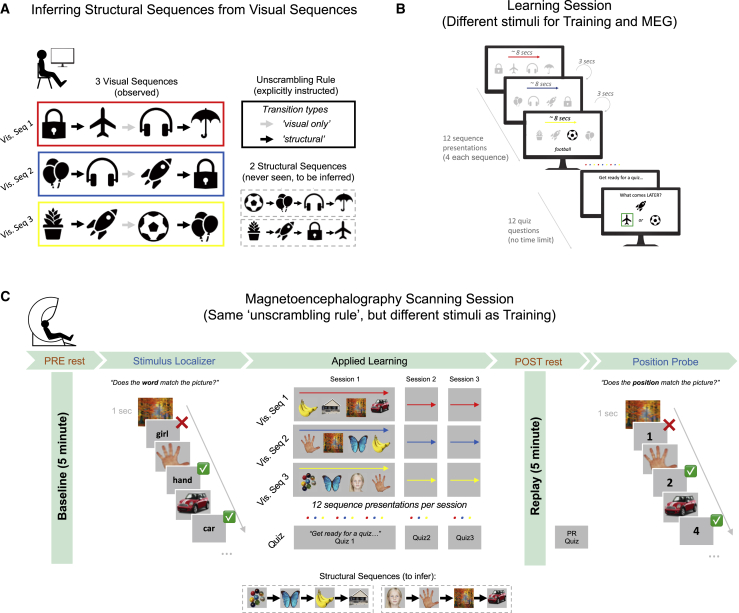
Task: Inferring structural relationships by applying an abstracted task template (A) Task outline. During pre-scan training, participants learned to infer correct “structural” relationships between eight pictures by passively observing three scrambled “visual” sequences and applying an explicitly instructed “unscrambling rule” (see main text). (B) Structure of learning session. In a single learning session, participants were shown each “visual sequence” four times and were instructed to mentally unscramble these observations to infer the correct sequential relationships using the learned “unscrambling rule.” Knowledge of the structural relationships between pairs of pictures was assessed in a quiz at the end of the session (Figure 2). Participants completed three learning sessions in both a pre-scan training visit (Structure Learning), and MEG visit (Applied Learning). (C) Structure of MEG scanning session. The MEG session began with a 5 min eyes-open rest session (pre-learning) followed by a Stimulus Localizer in which participants were repeatedly shown task pictures in a random order and had to indicate with a button press whether a word that followed each picture matched the preceding picture. Participants then completed three Applied Learning sessions (structurally identical to those experienced during pre-scan training, shown in (B), but with an entirely new picture set), followed by a second 5 min eyes-open rest session (post-learning). Knowledge was assessed in a quiz after each Applied Learning session (Q1, Q2, and Q3) and again following post-learning rest (PR quiz, see Figure 2). The scan finished with a Position Probe task, which differed from the Stimulus Localizer in that a number (1, 2, 3, or 4) followed each picture, instead of a word, and participants indicated whether the number matched the preceding picture’s inferred ordinal position. For quiz and Stimulus Localizer/Position Probe performance see Figures 2 and S1B, respectively.

Fifty-five participants (28 patients [13 unmedicated] and 27 healthy volunteers; Table S1) completed a structural inference task during MEG, in which they needed to infer correct sequential relationships (transitions) between eight task pictures by reorganizing their visual experience of these pictures (Figure 1). Participants were informed that the eight pictures were embedded in two “structural sequences,” each a linear chain with four states (i.e., [A→B→C→D] and [A′→B′→C′→D′]). Participants were also informed that they would never be shown a complete “structural sequence” from start to finish (e.g., [A→B→C→D]). Instead, they would passively observe three scrambled sequences of task pictures, each containing transitions from *both* “structural sequences” (e.g., [A′→B′→A→B]). We refer to these scrambled sequences as “visual sequences.” To complete the task, participants therefore needed to unscramble the “visual sequences” into the true “structural sequences.” This paradigm has previously been shown to induce spontaneous off-task neural replay of inferred transitions between pictures (i.e., task structure) (Liu et al., 2019).

**Figure S1:**
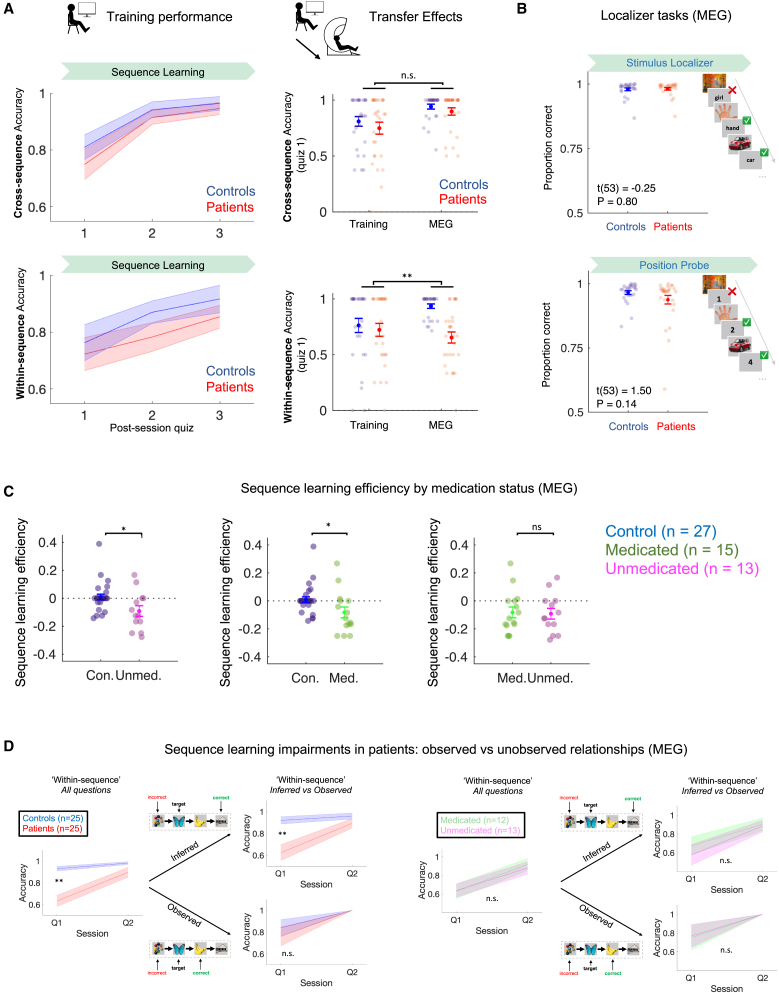
Pre-scan training performance, transfer effects, and additional MEG behavioral results, related to Figures 1 and 2 (A) During pre-scan training (visit 1) participants were explicitly instructed on the unscrambling rule, and completed 3 Structure Learning sessions with a different picture set to MEG (visit 2). (Left) Knowledge quiz performance following each Structure Learning session (1, 2 & 3), separately for cross-sequence and within-sequence questions (see Figure 2A for details). (Right) Behavioral transfer from training to MEG was assessed by comparing behavioral accuracy following the first Learning session (quiz 1) on visit 1(training) and visit 2 (MEG). For cross-sequence questions, training ^∗^ group ANOVA revealed a main effect of training (F(1,53) = 14.3, p = 0.0004), but no effects of group (F(1,52) = 1.50, p = 0.23) or interaction (F(1,53) = 0.05, p = 0.82, denoted by n.s.), indicating that patients and controls benefited equivalently from training on this structural component. By contrast, for within-sequence questions there was no main effect of training (F(1,53) = 1.40, p = 0.24), but significant effects of group (F(1,53) = 8.05, p = 0.006) and training ^∗^ group interaction (F(1,53) = 7.45, p = 0.009, denoted by ^∗∗^), indicating that patients were less able to transfer an abstracted sequential task template (cognitive map) from training to MEG, compared to controls. Similar statistical results obtain when using the learning lag estimates as the dependent variable. Patients (n = 28, red), controls (n = 27, blue). Error bars represent SEM. (B) Patients and controls are matched in performance during Stimulus Localizer (Left) and Position Probe (Right) tasks during MEG (Stimulus Localizer accuracy: patients = 98.1% ± 0.49, controls = 97.6% ± 0.54, t(53) = −0.25, p = 0.80. Position Probe accuracy: patients = 93.8% ± 1.70, controls = 96.6% ± 0.75, t(53) = 1.50, p = 0.14, two-tailed unpaired t test. Chance accuracy = 50%). See Figure 1C for session details. Patients (n = 28, red), controls (n = 27, blue). Error bars represent SEM. (C) Sequence learning efficiency by patient medication status. (Left) Control versus unmedicated patients, z = 2.42, p = 0.015. (Middle) Control versus medicated patients, z = 2.20, p = 0.028. (Right) Medicated versus unmedicated patients, z = 0.16, p = 0.872. Wilcoxon rank sum test for independent samples. Sample: control n = 27, unmedicated patient n = 13, medicated patient n = 15. Error bars represent SEM. (D) Quiz performance for ‘within-sequence’ questions, stratified by questions probing knowledge of a directly observed structural transition (e.g., A→B) versus an unobserved (i.e., inferred) transition (e.g., A→C). Analysis conducted in Q1 and Q2 only, so as to minimize participant exclusion for incomplete data. (Left) Patients show impaired sequence learning efficiency for *inferred* versus *observed* sequential relationships, compared to controls (Inferred relationships, group ^∗^ session ANOVA, main effect of session F(1,48) = 17.3, p = 1^∗^10^−4^. Main effect of group F(1,48) = 10.33, p = 0.002. Group ^∗^ session interaction F(1,48) = 9.51, p = 0.003, denoted by ^∗∗^. Observed relationships, group ^∗^ session ANOVA, main effect of session F(1,48) = 12.1, p = 0.001. Main effect of group F(1,48) = 0.48, p = 0.49. Group ^∗^ session interaction F(1,48) = 0.48, p = 0.49, denoted by n.s.). (Right) No significant difference between medicated and unmedicated patients in learning efficiency for inferred versus observed relationships (Inferred relationships, group ^∗^ session ANOVA, main effect of session F(1,23) = 16.5, p = 4^∗^10^−4^. Main effect of group F(1,23) = 0.31, p = 0.58. Group ^∗^ session interaction F(1,23) = 0.35, p = 0.56. Observed relationships, group ^∗^ session ANOVA, main effect of session F(1,23) = 7.28, p = 0.013. Main effect of group F(1,23) = 0.01, p = 0.92. Group ^∗^ session interaction F(1,23) = 0.01, p = 0.92, denoted by n.s.). Sample: controls, n = 25 (blue), patients, n = 25 (red), unmedicated patients, n = 13 (magenta), medicated patients, n = 12 (green). 5 participants (3 patients, 2 controls) excluded for incomplete data (i.e., participants who did not have at least one example of an ‘inferred’ and ‘observed’ question type in each quiz, owing to pseudorandom question selection at each quiz). These results remain similar when including Q3, albeit with 3 further participant exclusions for incomplete data.

Participants took part in two sessions. On visit 1 (pre-scan training), they were explicitly taught an “unscrambling rule” that determined how eight pictures, presented in three “visual sequences,” were embedded in two “structural sequences.” Namely, the first and last transitions of each “visual sequence” (i.e., [A′→B′] and [A→B], from [A′→B′→A→B]) reflected correct “structural relationships,” whereas the middle transition (i.e., [B′→A]) was a spurious (“visual only”) relationship (Figure 1A). Participants then practiced applying this rule in three Structure Learning sessions (∼5 min each) (Figures 1B and S1A).

On visit 2, participants completed three similar learning sessions during MEG scanning. The MEG task was conducted with eight entirely novel pictures but used the same unscrambling rule as pre-scan training (Figure 1C). This ensured that participants could not transfer specific knowledge about the structural embedding of any picture from visit 1 to visit 2 but instead needed to apply the previously learned *template* of the task structure, abstracted from sensory features, to the MEG session. Accordingly, we refer to the MEG learning sessions as “Applied Learning.” The Applied Learning task was immediately followed by a 5 min post-learning rest session, wherein we investigated group differences in spontaneous neural replay.

### Patients with schizophrenia show specific sequence-learning impairments

**Figure 2:**
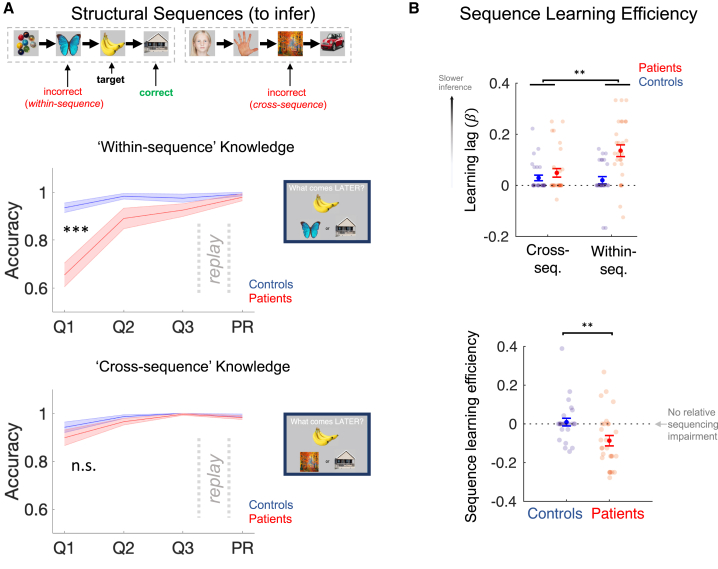
Patients with schizophrenia show specific sequence-learning impairments (A) (Top) Participants completed a knowledge quiz following each Applied Learning session (Q1, Q2, and Q3) and following the post-learning rest session (PR). Each question asked participants which of two probe pictures “come later” than a target picture in the “structural sequence.” We analyzed accuracy separately for questions that relied on knowledge of within-sequence picture order (i.e., incorrect probe picture preceded the target in the *same* ”structural sequence”) and for questions that could be answered using a process of elimination that did not require knowledge of sequential structure (i.e., cross-sequence questions in which the target and incorrect probe pictures belonged to *different* sequences). (Middle) Patients required more sessions than controls to attain ceiling level knowledge on within-sequence questions (group ^∗^ session ANOVA, main effect of session F(106,2) = 23.26, p = 4.20^∗^10^−9^; main effect of group F(53, 1) = 17.36, p = 0.0001; group ^∗^ session interaction F(106, 2) = 11.80, p = 2.37^∗^10^−5^, denoted by ^∗∗∗^). (Bottom) This group difference was absent for cross-sequence knowledge (main effect of session F(106,2) = 11.49, p = 3.05^∗^10^−5^; main effect of group F(53, 1) = 2.17, p = 0.15; group ^∗^ session interaction F(106, 2) = 0.70, p = 0.50, denoted by n.s.). Groups were matched for performance immediately prior to and following post-learning rest (quiz 3 within-sequence accuracy: patients = 92.6% ± 2.65 [mean ± SEM], controls = 97.5% ± 1.71, t(53) = 1.56, p = 0.12; quiz 3 cross-sequence accuracy: patients = 99.6% ± 0.40, controls = 100% ± 0.00, t(53) = 0.98, p = 0.33; PR quiz within-sequence accuracy: patients = 97.9% ± 1.46, controls = 99.3% ± 0.74, t(53) = 0.81, p = 0.42; PR quiz cross-sequence accuracy: patients = 98.3% ± 0.83, controls = 99.1% ± 0.64, t(53) = 0.78, p = 0.44, two-tailed unpaired t tests). (B) (Top) We defined a “learning lag” estimate for each participant and question type as the slope of a regression of quiz performance on session number. Group ^∗^ question-type ANOVA on this lag measure revealed a significant interaction effect (F(1,53) = 8.18, p = 0.006, denoted by ^∗∗^; main effects of group F(1,53) = 15.5, p = 0.0002 and question-type F(1, 53) = 5.36, p = 0.02). (Bottom) To capture this learning asymmetry, we defined sequence learning efficiency as lag_cross_ − lag_within_. Here, negative estimates indicate an impairment in inferring sequential relationships, beyond any participant-specific general cognitive impairment, which is expected to manifest in both cross- and within-sequence lag estimates. ^∗∗^ denotes p < 0.01 group difference (Wilcoxon rank sum test for independent samples, two tailed, see Results). Patients: n = 28 (red), controls: n = 27 (blue). Error bars represent group SEM. See Figure S1C for results by patient medication status.

We assessed the speed at which participants inferred the correct “structural sequences” by applying a brief knowledge quiz following each of three Applied Learning sessions and once more following the post-learning rest session. The quiz was designed so as to allow an assessment of the learning trajectories for both sequential (e.g., “banana” comes before “house”) and non-sequential (e.g., “house” and “hand” belong to different sequences) aspects of task structure and also provided a measure of this knowledge at the start of the post-learning rest session (Figures 1 and 2A).

Patient and control participants displayed matched knowledge of the “structural sequences” (i.e., the structural embedding of each picture) immediately prior to post-learning rest (i.e., quiz 3, following the final Applied Learning session; Figure 2A), as well as matched knowledge retention after this session (assessed both in a quiz immediately following post-learning rest, Figure 2A, and at the end of the MEG scanning session in a Position Probe task, Figures 1C and S1B).

However, patients showed a marked impairment in speed of knowledge acquisition, specific for the sequential component of task structure (see Figure 2 for details). To capture this, we defined a “sequence learning efficiency” measure that quantifies the speed of sequence learning for each participant, controlling for non-specific between-participant differences in behavior, such as those attributable to attention or rule comprehension (see Figure 2B for details). Sequence learning efficiency was significantly reduced in patients relative to controls (median patients = −0.13 (IQR −0.17 – 0.00, n = 28), controls = 0.00 (IQR −0.02 – 0.00, n = 27). *Z* = 2.81, p = 0.005. Wilcoxon rank sum test for independent samples, two tailed, Figure 2B), reflecting an impaired ability to benefit fully from the abstracted task template acquired during training (see Figure S1A for details), and was unrelated to working memory capacity measured before the scan (r(53) = −0.036, p = 0.79). Interestingly, the learning impairment in patients was particularly pronounced for associations requiring transitive inference (e.g., [A→ []→C]) as opposed to directly observed transitions (e.g., [A→B]; Figure S1D).

### Reduced spontaneous offline replay for inferred structure in patients

**Figure 3:**
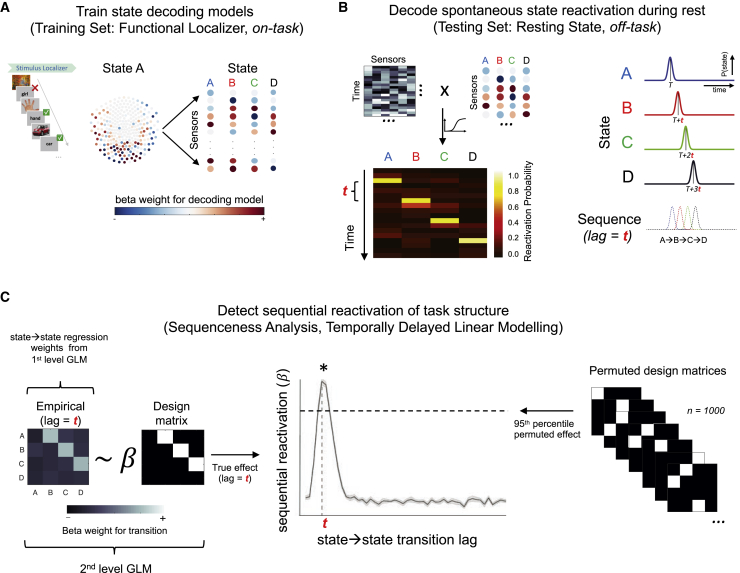
Sequential replay analysis pipeline (Temporally Delayed Linear Modeling, TDLM) (A) We trained separate multivariate decoding models for each task stimulus using MEG sensor data acquired in Stimulus Localizer. (B) We next applied peak-accuracy decoders to neural data from the rest scan to derive a decoded [time × state] reactivation matrix. (C) Finally, we quantified the evidence that spontaneous neural reactivations sequentially replay the inferred task structure (e.g., A_T_→B_T+t_) using a two-step lagged regression approach (TDLM). We estimated the magnitude of this effect independently at each transition lag (t={10 ms, 20 ms, …, 600 ms}) and used non-parametric permutation tests to define a FWE-corrected significance threshold (see STAR Methods for details).

Having identified a specific sequence learning impairment in patients, we next investigated whether patients also exhibited impaired spontaneous neural replay of task structure, after learning. Analogous to protocols in rodent replay experiments (Ji and Wilson, 2007; Wilson and McNaughton, 1994), the final Applied Learning session was immediately followed by a rest session (5 min post-learning rest, eyes open; Figure 1C). To detect spontaneous sequential reactivation of task states during this session (i.e., offline replay), we adopt the Temporally Delayed Linear Modeling (TDLM) framework (Figure 3) (Liu et al., 2021a, 2019; Wimmer et al., 2020).

**Figure S2:**
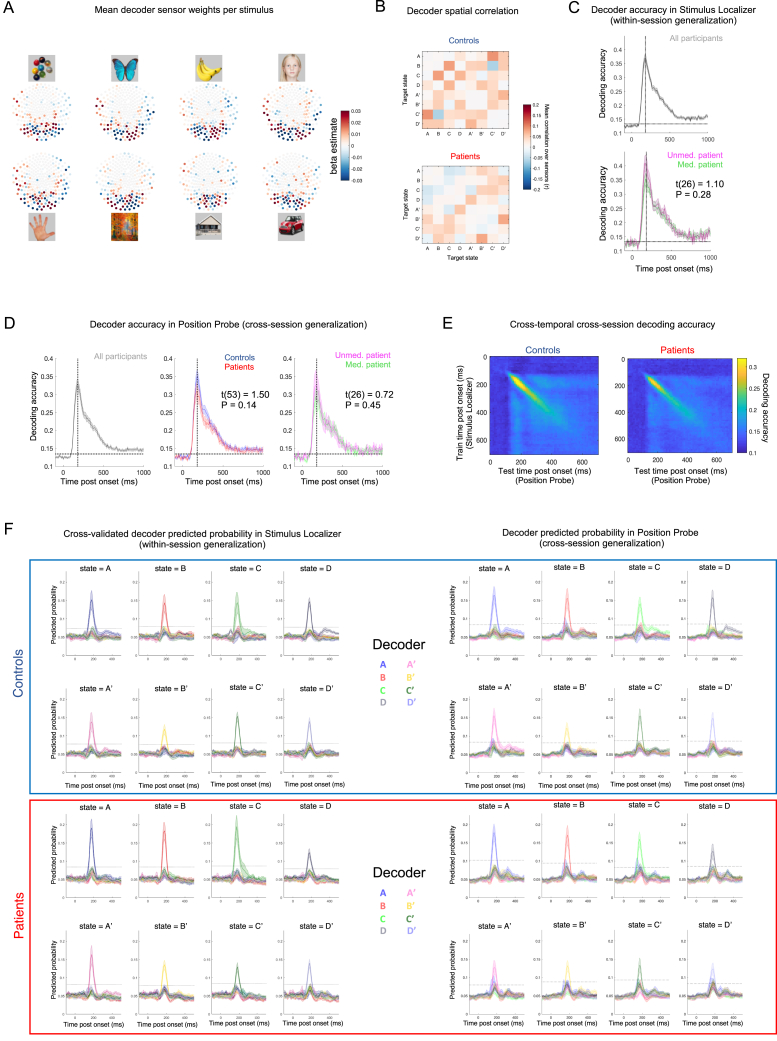
Sensor maps, spatial correlation, and performance for decoding models, related to Figure 3 and 4 (A) Mean regression weight on each MEG sensor for trained decoding model of each task picture (mean over combined sample, for illustration only. Top represents front of head). (B) Mean within-subject Pearson’s correlation coefficient between sensor weights of trained decoders (i.e., spatial correlation) for each state pair. (C) Cross-validated decoding accuracy of decoders trained and tested on neural data from Stimulus Localizer (i.e., within-session generalization. Abscissa represents time point of decoder training and testing). Plotted for combined sample (Top) and for patients stratified by D2/3 antagonist medication status (Bottom, no group difference at 180 ms, t(26) = 1.10, p = 0.28, two-tailed unpaired t test). (D) Decoding accuracy for decoders trained on neural data from Stimulus Localizer and tested on neural data from Position Probe (i.e., cross-session generalization. Abscissa represents time point of decoder training and testing). Plotted for combined sample (Left), patients versus controls (Middle, no group difference at 180 ms, t(53) = 1.50, p = 0.14, two-tailed unpaired t test), and for patients stratified by D2/3 antagonist medication status (Right, no group difference at 180 ms, t(26) = 0.72, p = 0.45, two-tailed unpaired t test). For (C) & (D) vertical dashed line at 180 ms, horizontal dashed line at peak-level P_FWE_ = 0.05 significance threshold derived from test data label permutation. (E) Cross-session decoding accuracy at each combination of decoder training time (Stimulus Localizer data) and testing time (Position Probe data), plotted for patients (right) and controls (left) separately. (F) Mean (reactivation) probability estimate of decoders (target state of decoder coded by line color) for test data corresponding to each state (different ground truth state in each panel). Decoders trained on neural data from 180 ms post-stimulus onset (Stimulus Localizer data) and tested at each time point following stimulus onset (plotted on abscissa). (Left) Within-session decoding performance (Stimulus Localizer test data from left-out sample in cross-validation), (Right) Cross-session decoding performance (Position Probe test data). Horizontal dashed lines represent group-specific peak-level P_FWE_ = 0.05 significance threshold for the correct (i.e., peaked) decoder, derived from test data label permutation, and controlling for multiple comparisons over time points. Plotted for controls (Top) and patients (Bottom) separately. Samples for (B) – (F): patients (n = 28, red), controls (n = 27, blue). (C) & (D): Medicated patients (n = 15, green), un-medicated patients (n = 13, magenta). (A): n = 53 (2 participants used slightly different picture set). Error bars represent SEM.

**Figure 4:**
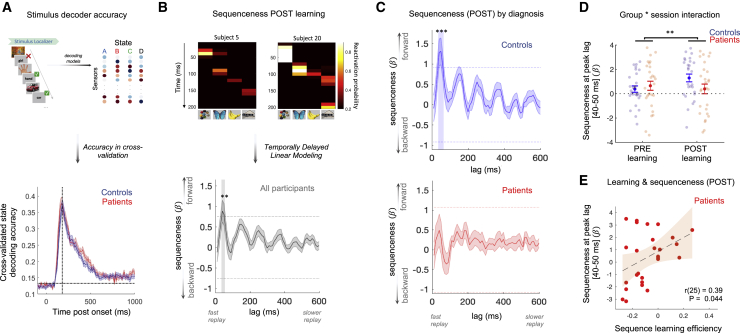
Reduced spontaneous offline replay for inferred structure in patients (A) Cross-validated decoding accuracy of neural state decoders trained and tested on data from Stimulus Localizer (train and test on same time bin post-onset, abscissa). Vertical dashed line at peak decoding accuracy across participants (180 ms). Horizontal dashed line at peak-level P_FWE_ = 0.05 threshold for chance-level accuracy (test-data label permutation). Patients: n = 28 (red), controls: n = 27 controls (blue). See Figure S2 for non-significant medication effects and cross-session generalization performance. (B) (Top) [Time × state] reactivation matrices (from post-learning rest, POST) from two control participants, showing two exemplar forward replay events. (Bottom) Sequenceness effect (*forward – backward* replay) in combined sample during post-learning rest, showing a significant effect for a predominant forward replay of task structure (e.g., A→B) at 40 and 50 ms transition lag (shaded patch, used as temporal region of interest for subsequent analyses, e.g., D and E). Horizontal dashed lines represent peak-level P_FWE_ = 0.05 significance thresholds derived from state label permutation at 2^nd^-level design matrix of TDLM (see Figure 3 and STAR Methods). ^∗∗^denotes P_FWE_ < 0.01, n = 54 (n = 27 each group). (C) Significant forward sequenceness post-learning (POST) in control participants (top, blue, n = 27) at 40–60 ms lags (shaded patch, P_FWE_ < 0.05) but not in patients (bottom, red, n = 27). Horizontal dashed lines as in (B). ^∗∗∗^ denotes P_FWE_ < 0.001. (D) Group ^∗^ session interaction in sequenceness (effect at significant time lags, from B). ^∗∗^ denotes p < 0.01 (group ∗ session ANOVA, interaction effect). Error bars in (A–D) represent group SEM. (E) Significant linear relationship between peak post-learning (POST) sequenceness and sequence learning efficiency in patients. Shaded patch designates 95% confidence intervals for line of best fit. See Figure S3 for non-significant effects of medication, replay associated with pre-learning rest and “visual-only” transitions, and for non-significant MEG-learning correlation in controls.

We first used visually evoked neural data from a pre-learning Stimulus Localizer (Figure 1C) to train a multivariate decoding model for each picture (i.e., eight one-versus-rest lasso-regularized logistic regression models at each 10 ms sample of the evoked response; Figures 3A and S2). As Stimulus Localizer preceded Applied Learning sessions, the decoder training data carried no information pertaining to task structure (Liu et al., 2021a, 2019). The localizer included an incidental task to encourage attentional maintenance, which was matched between groups (performance in patients = 98.1% ± 0.49 [mean ± SEM], controls = 97.6% ± 0.54, t(53) = −0.25, p = 0.80, two-tailed unpaired t test; Figure S1B). Decoding accuracy was assessed for each participant and time point following stimulus onset in leave-one-out cross validation (“train” and “test” data both from Stimulus Localizer). Across all participants, peak decoding accuracy was achieved at 180 ms after stimulus onset and was matched between groups (patients: 36.4% ± 2.11, controls: 37.2% ± 1.75, t(53) = 0.17, p = 0.87, two-tailed unpaired t test; chance ∼12.5%; Figures 4A and S2C). We additionally assessed the ability of decoders trained on neural data from Stimulus Localizer (start of scan) to accurately classify unseen data from a similar Position Probe session (end of scan; Figure 1C), finding no group accuracy difference at the time point of peak decodability (t(53) = 1.50, p = 0.14, two-tailed unpaired t test; Figure S2D).

Having confirmed equivalent decoding accuracy between groups, we next applied the trained decoders to MEG data from the post-learning rest session for each participant, allowing us to estimate the spontaneous reactivation probability for each task picture at each time point (decoders trained at time bin of group peak decoding accuracy; Liu et al., 2019; Wimmer et al., 2020; Figures 3B and 4B). We then quantified the evidence for sequential replay of task structure in the rest session as a whole for a given transition lag (from 10 ms to 600 ms) using a two-stage regression approach; first quantifying the unique evidence for each pairwise state→state transition in the neural reactivation matrix, and then assessing the degree to which the pattern of the these effects (across all state pairs) corresponded to replay of the inferred task structure (Figure 3C) (Liu et al., 2021a, 2019; Wimmer et al., 2020). We defined “sequenceness” as an asymmetry between replay in a forward (e.g., [A→B]) and backward (e.g., [B→A]) direction (Kurth-Nelson et al., 2016; Liu et al., 2019).

**Figure S3:**
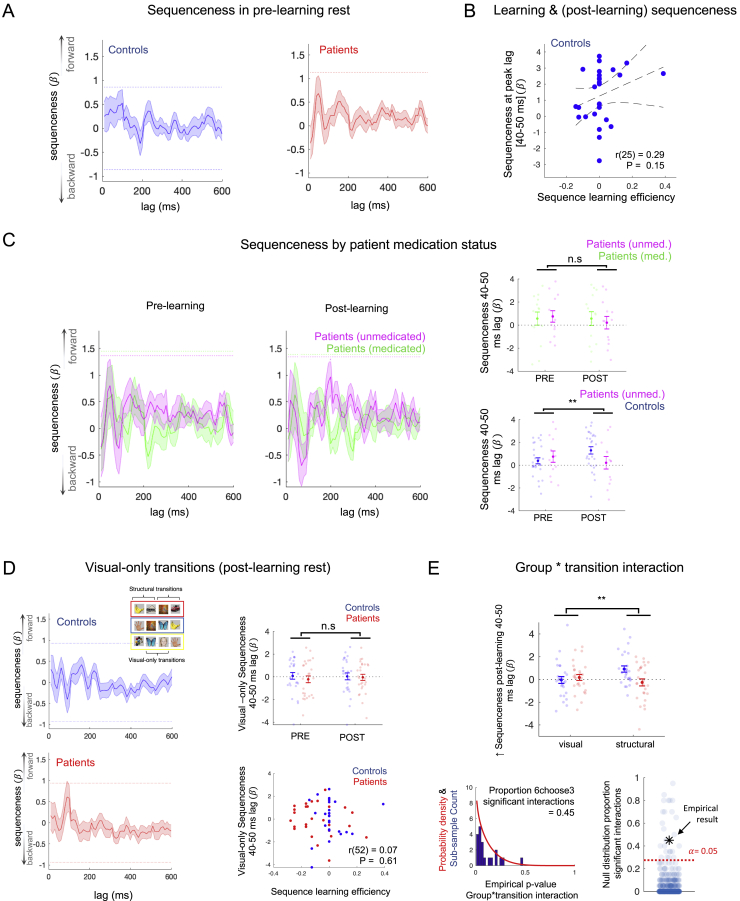
Sequenceness pre-learning, by medication status, and for visual-only transitions, related to Figure 4 (A) As a control analysis we quantified sequenceness during pre-learning rest, prior to stimulus observation. We plot the non-significant effect separately for controls (Left) and patients (Right), at state→state transition lags form 10 – 600 ms. (B) Non-significant correlation between sequence learning efficiency and post-learning sequenceness (40 – 50 ms lag) in control participants (r(25) = 0.29, p = 0.15). See Results and Figure 4 for result in patients and combined sample. Error bars represent 95% confidence intervals for line of best fit. (C) (Left & Middle) Sequenceness pre- and post-learning plotted for patients taking D2/3 medication, and those not taking medication. (Right, top) There were no significant effects (main or interaction) in a group ^∗^ session mixed ANOVA comparing peak sequenceness (40 – 50 ms lag) in patients stratified by medication status (group ^∗^ session ANOVA, group: F(1,25) = 0.01, p = 0.91, session: F(1,25) = 0.75, p = 0.39, interaction: F(1,25) = 0.75, p = 0.39, denoted by n.s.). (Right, bottom) The patient versus control group ^∗^ session interaction on peak sequenceness (40 – 50 ms lag) remained significant when restricting the patient sample to those not taking D2/3 antagonist medication (group ^∗^ session ANOVA, group: F(1,38) = 0.52, p = 0.48, session: F(1,38) = 0.59, p = 0.45, interaction: F(1,38) = 8.81, p = 0.005, denoted by ^∗∗^). (D) (Left) Sequenceness (post-learning rest) for visual-only transitions (i.e., those that appear in the visual sequences during Applied Learning, but do not contribute to inferred structural sequences, see Figure 1A) plotted for controls (top) and patients (bottom) separately. (Right, top) There was no effect of learning on visual sequenceness extracted at 40 – 50 ms lag (session ^∗^ group mixed ANOVA, session: F(1,52) = 0.11, p = 0.75, group: F(1,52) = 0.29, p = 0.59, interaction: F(1,52) = 0.31, p = 0.58, denoted by n.s.). (Right, bottom) There was no relationship between visual sequenceness (at 40 – 50 ms lag) post-learning and sequence learning efficiency (r(52) = 0.07, p = 0.61). For (A), (C), and (D) horizontal dashed lines in all sequenceness plots represent group-specific peak-level P_FWE_ = 0.05 significance thresholds derived from state label permutation at 2nd-level design matrix of Temporally Delayed Linear Modeling, controlling for multiple comparisons across lags. (E) (Top) Applied Learning resulted in a greater increase in sequenceness for structural versus visual transitions in controls relative to patients (group ^∗^ transition mixed ANOVA on the post-learning increase in sequenceness at 40 – 50 ms lag revealed a significant interaction: F(1,52) = 6.80, p = 0.01, denoted by ^∗^, with no main effect of transition: F(1,52) = 0.97, p = 0.33, or group: F(1,52) = 2.46, p = 0.12). Control participants demonstrated a significant difference between learning-induced change for structural versus visual transitions (t(26) = 2.78, p = 0.011, one-sample t test, 2 tailed), whereas patients did not (t(26) = −1.07, p = 0.29, one-sample t test, 2 tailed). (Bottom) To ensure that the significant group ^∗^ transition interaction was not confounded by the different number of structural (n = 6) and visual (n = 3) transitions in the task, we recalculated the interaction effect (E, Top) for all possible subsamples of n = 3 structural transitions (i.e., 6 choose 3 = 20 subsamples). (Bottom, left) 45% of the ‘6 choose 3' subsample analyses yielded an interaction effect p < 0.05 (histogram of subsample counts and fitted probability density beta distribution shown. y axis scale pertains to counts). (Bottom, right) The observed proportion of significant interactions (45%) is higher than 95% of the observed ‘significant proportion’ effects from a null distribution of simulated datasets instantiating the hypothesis that no group ^∗^ transition interaction effect exists (red dashed line, derived from 500 simulated experiments of n = 20 subjects per group. Simulation code adapted from https://github.com/matthewnour/TDLM_alpha_simulation/). Sample for all analyses: patients (n = 27, red. [Medicated n = 14, green. Unmedicated n = 13, magenta]), controls (n = 27, blue). Error bars (except B) represent SEM. PRE and POST denote pre- and post-learning rest sessions, respectively.

In the post-learning rest period, we found evidence for forward sequenceness associated with a peak transition lag of 40–50 ms in the combined sample of patients and controls, replicating previous findings (Liu et al., 2019) (maximal effect at 40 ms lag: β = 0.89 ± 0.27, P_FWE_ = 0.005, peak-level significance threshold derived from a non-parametric permutation test, family-wise error (FWE) corrected across lags, n = 54; Figure 4B). Analyzing patient and control samples separately indicated that control participants alone exhibited statistically significant forward sequenceness (significant lags: 40–60 ms, maximal effect at 50 ms lag: β = 1.31 ± 0.33, P_FWE_ < 10^−3^, n = 27). The patient sample did not show significant sequenceness at any time lag (maximal [non-significant] effect at 40 ms lag: β = 0.49 ± 0.41, P_FWE_ = 0.80, n = 27; Figure 4C). Of note, the distribution of peak-effect time lags was similar in patient and control samples (median in controls = 40 ms [IQR = 22.5–70.0] and patients = 50 ms [20.0–67.5]; *Z* = −0.28, p = 0.78; Wilcoxon rank sum test for independent samples, two tailed; median lag of maximal absolute sequenceness in range 0–300 ms). As a control analysis, we quantified sequenceness in the pre-learning rest session (neural data with the same statistical and oscillatory properties as post-learning rest, but devoid of any structural replay), finding no suprathreshold effect in either group (Figure S3A).

We contrasted the peak sequenceness effect measured in pre- and post-learning rest. This contrast quantifies experience-induced spontaneous replay, controlling for participant-specific variance in statistical properties of rest data (Genzel et al., 2020; Ji and Wilson, 2007; Wilson and McNaughton, 1994). Crucially, this contrast was greater for controls compared to patients (group ^∗^ session mixed ANOVA, interaction: F(1,52) = 8.08, p = 0.006; main effects of group: F(1,52) = 0.52, p = 0.48, and session: F(1,52) = 2.51, p = 0.12; sequenceness extracted at 40–50 ms lag; Figure 4D). This interaction effect remained significant when excluding patients taking D2/3 antagonist medication (F(1,38) = 8.81, p = 0.005; Figure S3C).

There was a positive correlation between sequence learning efficiency and peak post-learning sequenceness in the patient group (r(25) = 0.39, p = 0.044; Figure 4E; sequenceness extracted at 40–50 ms lag). Although this correlation was not significant in control participants alone (who displayed relatively little behavioral variance, r(25) = 0.29, p = 0.15; Figure S3B), there was a significant behavior-MEG relationship in the combined sample of patients and controls (sequenceness ∼ group ^∗^ sequence learning efficiency regression, β_learning_ = 5.13 ± 2.10, t(50) = 2.44, p = 0.018), which did not significantly differ between groups (β_interaction_ = −0.10 ± 4.19, t(50) = −0.24, p = 0.81. β_group_ = 0.39 ± 0.53, t(50) = 0.74, p = 0.46). A similar regression analysis showed that sequence learning efficiency also predicted a *boosting* in peak neural replay following learning (post-learning minus pre-learning sequenceness, β_learning_ = 3.74 ± 1.73, t(50) = 2.16, p = 0.035), with no effect in a control analysis using sequenceness from pre-learning rest (β_learning_ = 1.39 ± 1.98, t(50) = 0.70, p = 0.49). Given that knowledge of “structural sequences” was equivalently high in both groups immediately prior to, and after, post-learning rest (Figure 2A), these findings suggest a tentative hypothesis that the strength of spontaneous offline replay might reflect the strength of an abstracted neural template of task structure present during learning, which facilitates assimilation of new experiences into an existing cognitive schema (Liu et al., 2019). Evidence that such a template exists in control participants to a greater degree than patients is found in the behavioral transfer effects from training to MEG, which show a significant group difference (see Figure S1A for details).

We found no evidence for sequenceness corresponding to “visual only” transitions, which are present in visually observed, but not structurally inferred, sequences (Figure S3D). Crucially, an increase in sequenceness following learning was significantly greater for structural versus visual transitions in control participants alone (group ^∗^ transition ANOVA on post-learning minus pre-learning sequenceness reveals a significant interaction effect: F(1,52) = 6.80, p = 0.01, with no main effect of transition: F(1,52) = 0.97, p = 0.33, or group: F(1,52) = 2.46, p = 0.12; Figure S3E), consistent with the notion that spontaneous replay reflects inferred relationships as opposed to merely observed transitions (Gupta et al., 2010; Liu et al., 2019). Moreover, there was no relationship between “visual only” sequenceness and sequence learning efficiency (r(52) = 0.07, p = 0.61; Figure S3D).

**Figure S4:**
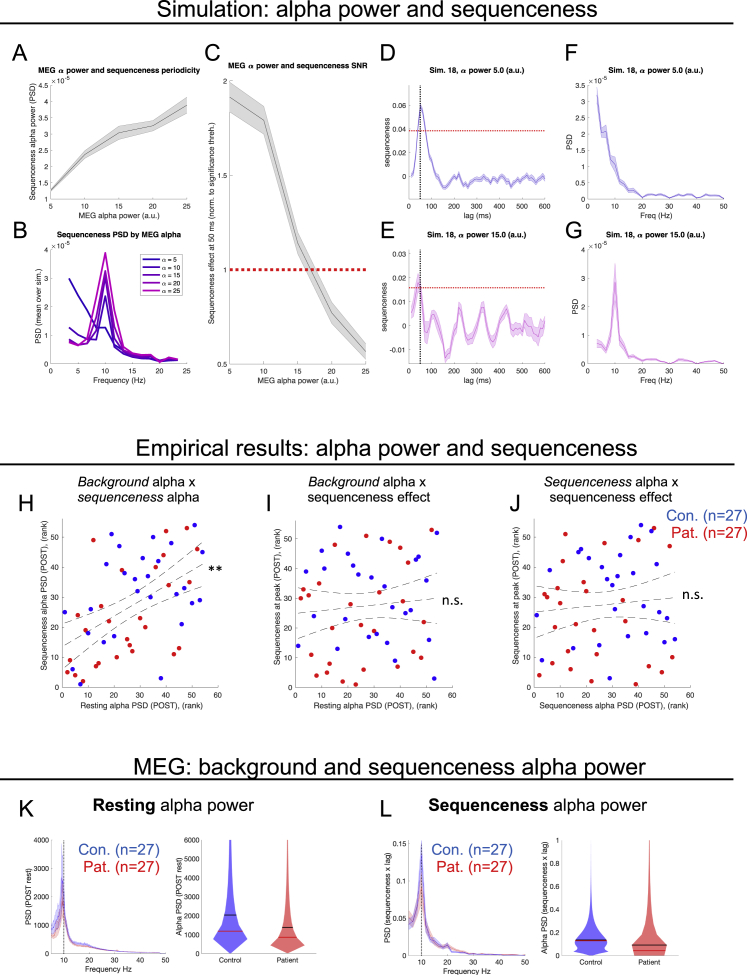
Exploration of influence of background alpha oscillation in rest data, related to Figure 4 (A–G) Simulations, illustrating effect of background MEG alpha oscillation on sequenceness periodicity and signal-noise ratio (SNR). *Details of simulation:* For each simulated participant we generated 1 min of synthetic MEG data (272 sensors, sampled at 100 Hz, incorporating cross- and auto-correlation sensor relationships). Synthetic data contained (1) forward sequential reactivations of sensor patterns for 8 states at 50 ms lag, in a manner that replays a hypothetical task structure, as hypothesized in our task, and (2) an additive alpha-band amplitude modulation to the MEG time series (frequency ~ N(μ=10, σ=0.2)). We simulated 25 experiments (each of 20 participants) for 5 alpha ‘strength’ parameter levels, ranging from minimal (5 arbitrary units, a.u.) to extreme (25 a.u.). As in the main paper, for each experiment we run the full TDLM pipeline for each participant, controlling for 10 Hz oscillation, before averaging the *sequenceness x lag* effect over participants. For each experiment we then estimated (1) the power spectral density (PSD) of the group mean *sequenceness x lag* effect (using a discrete Fourier transform), and (2) the effect magnitude of the forward sequenceness effect at 50 ms lag (i.e., the ground truth effect). Code available at https://github.com/matthewnour/TDLM_alpha_simulation/ (A) Relationship between background alpha power and alpha-band periodicity (10 Hz PSD) of *sequenceness x lag* effect (Mean ± SEM over n = 25 experiments). Increased background alpha strength is reflected in increased alpha-band periodicity in sequenceness effects. (B) Mean power spectra of *sequenceness x lag* effect as a function of background alpha strength (each line is the mean of all subjects and all experiments). (C) Relationship between background alpha power and signal-to-noise ratio (SNR) for ground truth forward sequenceness effect at 50 ms lag (mean ± SEM over n = 25 experiments, effect normalized to experiment-specific significance threshold derived by permutation testing (Liu et al., 2019), marked by dashed horizontal line). Increased background alpha strength is reflected in a decreased power to detect a true sequenceness effect, if present. (D and E) Exemplar group-mean *sequenceness x lag* effect from a single low- and high-alpha experiment, respectively (mean ± SEM over n = 20 participants). (F and G) Corresponding power spectra for D & E. (H–J) Empirical results (POST-learning rest). Error bars represent 95% confidence intervals for line of best fit. (H) Consistent with simulation predictions (A), there is a positive correlation between alpha periodicity of *sequenceness x lag* effect, and background alpha power across subjects in MEG (Combined sample: rho(52) = 0.51. p < 0.001 [denoted by ∗∗]. Patients: rho(25) = 0.58, p = 0.002. Controls: rho(25) = 0.38, p = 0.05). (I) No significant correlation between background alpha power and peak sequenceness effect at 40-50 ms lag (Combined sample: rho(52) = 0.09. p = 0.50 [ns]. Patients: rho(25) = 0.19, p = 0.34. Controls: rho(25) = −0.07, p = 0.71). (J) No significant correlation between *sequenceness x lag* alpha periodicity and peak sequenceness effect at 40-50 ms lag (Combined sample: rho(52) = 0.09. p = 0.52 [ns]. Patients: rho(25) = 0.01, p = 0.96. Controls: rho(25) = −0.02, p = 0.91). All scatterplots display ranked effects, and correlations assessed with Spearman’s rank correlation coefficient. (K and L) Group differences in background MEG alpha power and sequenceness alpha power. (K) (Left) Power spectrum of MEG data during POST-learning rest session (Mean ± SEM over subjects) for patients (red, n = 27) and controls (blue, n = 27). (Right) Corresponding alpha (10 Hz) PSD for controls (n = 27) and patients (n = 27), with no significant difference (t(52) = 1.17, p = 0.25). Of note, there was also no significant group difference in resting alpha power measured during PRE-learning rest (t(52) = 0.85, p = 0.40), and group differences in resting alpha power remain non-significant (p > 0.35) when defining the alpha band as 8-13 Hz. (L) (Left) Power spectrum of *sequenceness x lag* effect during POST-learning rest (Mean ± SEM over subjects. Patients, red, n = 27, and controls, blue, n = 27). (Right) Corresponding alpha (10 Hz) PSD for controls (n = 27) and patients (n = 27), with no significant difference (t(52) = 1.56, p = 0.12). For spectral analyses, we used a discrete Fourier transform to calculate *sequenceness x lag* (simulation and empirical results: A, B, F – H, J, L) and *background MEG* (empirical results: H, I, K) spectrograms. For background alpha power we applied the Fourier transform to MEG time series from each channel separately, and calculated the mean power spectrum over channels, for each participant and session (sampling rate 400 Hz, bad-samples set to zero, bad channels excluded, power spectra smoothed with 2.5 Hz moving mean window). Alpha defined as 10Hz (dotted vertical lines). For violin plots we use normal kernel density estimation. Black and red horizontal lines indicate sample mean and median, respectively.

Finally, in supplementary analyses, we demonstrate that the oscillatory component in the group mean sequenceness plots (Figures 4B and 4C) is likely a result of a prominent background alpha oscillation characteristic of resting state data. The reported group difference in sequenceness is not attributable to alpha power differences, nor does this feature inflate the false positive rate in sequenceness analyses (see Figure S4).

### Replay-associated ripple power colocalizes to hippocampus and is augmented in patients

**Figure 5:**
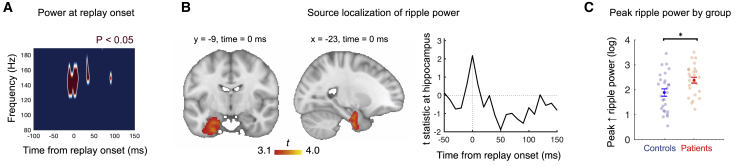
Replay-associated ripple power colocalizes to hippocampus and is augmented in patients (A) Replay onsets were identified as time bins during post-learning rest exhibiting >95^th^ percentile evidence for state→state reactivation of task structure at 40 ms lag, preceded by a low reactivation pre-onset baseline (see STAR Methods). Time-frequency decomposition of sensor-level data shows transient increase in high-frequency oscillatory power at replay onsets (averaged across sensors) during post-learning rest (red: p < 0.05, mass-univariate two-tailed one-sample t test versus pre-onset baseline −100 to −10 ms, n = 53 [26 patients and 27 controls], smoothed with 20 ms kernel for display). (B) (Left and Middle) Source localization of replay-associated 120–150 Hz power during post-learning rest (*a priori* spectral region of interest; Liu et al., 2019). T values for voxels showing positive relationship with subject-specific ripple power at replay onset (neurological orientation). Cluster significant at P_FWE_ < 0.05 (whole-brain corrected using non-parametric permutation test). (Right) Effect time-course from a peak hippocampal voxel identified in a previous fMRI study (Garvert et al., 2017) to represent an inferred relational structure, Montreal Neurological Institute [−15, −13, −19] (n = 53 [26 patients and 27 controls]. Group-level regression model controls for group differences in replay-associated ripple power, see STAR Methods). (C) Group difference in peak power increase extracted in range 120–150 Hz, following replay onset (replay epoch = 0 to 50 ms following onset, ± 10ms) during post-learning rest. ^∗^ denotes p < 0.05 (two-tailed unpaired t test). Patients: n = 26 (red), controls: n = 27 (blue). Error bars represent group SEM. See Figure S6A for results by medication status.

To investigate replay-associated (high-frequency) ripple power and localize its neural source, we identified onsets of putative replay events during post-learning rest as time points exhibiting high (>95^th^ percentile) evidence for sequential state reactivation for inferred task structure at 40 ms lag (lag of peak effect in the combined sample, Figure 4B; see STAR Methods) (Liu et al., 2019). Time-frequency decomposition for epochs centered on these onsets revealed a transient increase in ripple power (versus a low-reactivation pre-onset baseline, −100 ms to −10 ms), an increase significant across all participants in a region-of-interest analysis informed by our previous study (Liu et al., 2019) (120–150 Hz at onset ± 10 ms, 0.32 ± 0.12, t(52) = 2.6, p = 0.01, two-tailed one-sample t test; Figure 5A). As a control, we repeated this analysis for pre-learning rest, finding no evidence of transient ripple power increase (0.02 ± 0.10, t(52) = 0.21, p = 0.84, two-tailed one-sample t test). Source reconstruction of 120–150 Hz power at replay onset during post-learning rest, computed with a linearly constrained minimum variance (LCMV) beamforming algorithm (Liu et al., 2019; Van Veen et al., 1997), revealed a single cluster within the left medial temporal lobe predicted by subject-specific replay-associated ripple power in the combined sample of patients and controls (P_FWE_ < 0.05, whole brain non-parametric permutation test, no group difference) (Figure 5B).

Genetic mouse models of schizophrenia report increased resting hippocampal ripple power (Altimus et al., 2015; Suh et al., 2013; Zaremba et al., 2017). Consistent with these results, we found that the peak ripple power increase following replay onset was significantly higher in patients compared to controls (2.69 ± 0.12 versus 1.89 ± 0.15, t(51) = −2.6, p = 0.01, two-tailed unpaired t test; Figure 5C; peak ripple power extracted in range 120–150 Hz, in the time interval spanning a replayed transition during post-learning rest). One caveat is that our analysis approach could introduce a bias toward underestimating the replay-conditional ripple power in patients, where we find reduced evidence for sequential replay. This is an inherent limitation of probabilistic approaches to replay detection, but note that this limitation would, if anything, reduce our ability to detect the reported effect.

In exploratory analysis, we asked whether the temporal dynamics of spontaneous ripple “events,” defined independent of replay probability, differ between groups (defining an “event” as any time sample exceeding two SD of median ripple power measured over all sensors). We found no effect of session (pre- versus post-learning rest) or diagnosis on event rate (session: F(1,52) = 2.07, p = 0.16; diagnosis: F(1.52) = 0.29, p = 0.59; interaction: F(1,52) = 0.01, p = 0.90), lifetimes (i.e. median duration of contiguous suprathreshold events; session: F(1,52) = 3.24, p = 0.08; diagnosis: F(1.52) = 0.30, p = 0.59; interaction: F(1,52) = 1.78, p = 0.19) or interval times (i.e. median duration separating two non-contiguous events; session: F(1,52) = 1.52, p = 0.22; diagnosis: F(1.52) = 1.38, p = 0.24; interaction: F(1,52) = 3.35, p = 0.07; all results from session ^∗^ group ANOVA). We caution against an overinterpretation of these results, however, as MEG is relatively insensitive to high-frequency power originating from deep sources.

### Patients exhibit impaired reactivation of inferred relationships during high co-activation epochs

**Figure 6:**
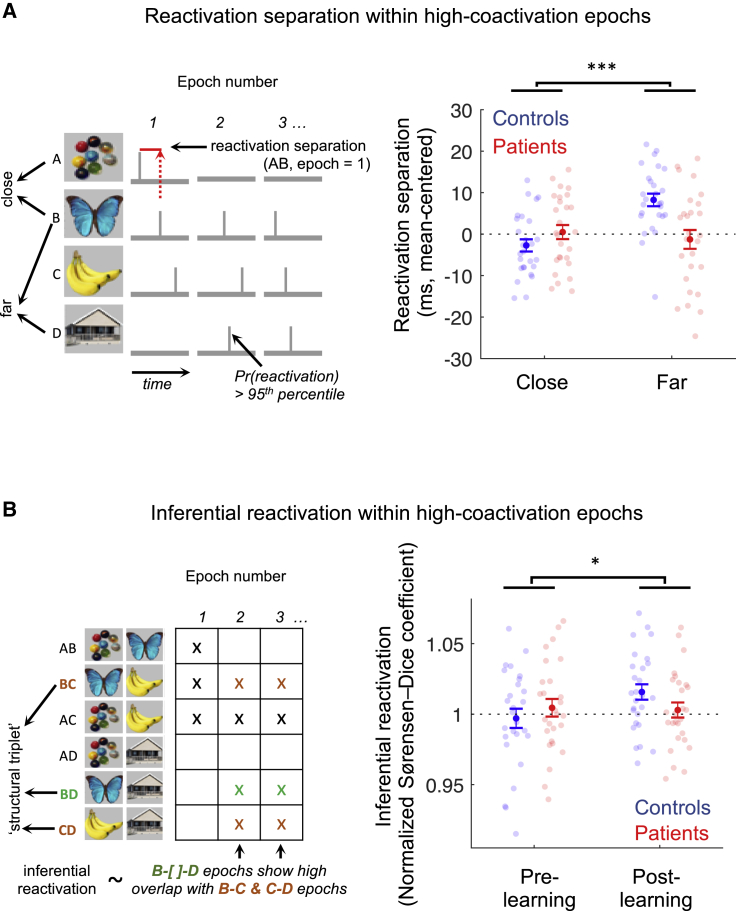
Patients exhibit impaired reactivation of inferred relationships during high co-activation epochs (A) High co-activation epochs were defined as 400 ms time windows centered on time bins of high instantaneous state reactivation probability (>95^th^ percentile summed decoder probability), preceded by a low reactivation pre-onset baseline. (Left) We define the reactivation separation for a pair of states as the median time separating their respective suprathreshold decoder reactivations within high-coactivation epochs (threshold = 95^th^ percentile of subject- and decoder-specific reactivation probability distribution). We compare this effect for structurally adjacent states (e.g., A and B, “close”) versus states separated by a single intermediate state (e.g., B and D, “far”) in each participant. (Right) Significant group ^∗^ distance ANOVA interaction in reactivation separation during post-learning rest (p < 0.001, denoted by ^∗∗∗^). Controls (n = 27, blue) exhibit a greater reactivation separation for “far” versus “close” pairs, whereas patients (n = 27, red) do not show this effect. See Figure S6C for results by medication status. (B) (Left) Within the same high-coactivation epochs (in A), we define an “inferential reactivation” effect that quantifies the degree to which reactivations representing a transitive inference (B-[]-D, unobserved) occur *preferentially* in the presence of their shared state (C), capturing the putative function of spontaneous reactivation in “stitching together” associative memories. For each sequentially related triplet (e.g., B-C-D, a *structural* triplet) we quantify the overlap between (1) epochs showing reactivation of the two states related by an inferred, but unobserved, transitive association (e.g., B and D) and (2) epochs showing reactivation of all three states (e.g., B and C and D). To capture the specificity of this reactivation pattern for *structural*-triplets, we operationalize “inferential reactivation” as the mean overlap effect over all *structural*-triplets, normalized by the effect over all *unique* triplets (e.g., A-A′-B, A-A′-C,…), for each participant (effects > 1 indicate positive inferential reactivation). (Right) Control participants (n = 27, blue) exhibit a greater increase in inferential reactivation after learning, compared to patients (n = 27, red; session ^∗^ group ANOVA interaction effect, p < 0.05, denoted by ^∗^). Error bars represent group SEM.

In rodents, SWRs and replay events are characterized by transient bursts of coincident place cell firing (i.e., neural state co-activations) (Barron et al., 2020; Buzsáki, 2015; Ji and Wilson, 2007; Wilson and McNaughton, 1994). Augmented ripple power in patients motivated us to ask whether patients might display a replay signature during analogous “high co-activation” epochs using an analysis that relaxes a requirement for replay sequences to occur at a specific transition lag (see Figure 6A) (Suh et al., 2013). In control participants, the median time separating reactivations was shorter for structurally adjacent states (e.g., A-B) compared to states pairs separated by an intermediate state (e.g., A-C). This contrast was significantly greater to that seen in patients, convergent with rodent findings (Suh et al., 2013) (group ^∗^ distance ANOVA, interaction effect: F(1,52) = 12.2, p = 0.001; main effects of group: F(1,52) = 3.41, p = 0.07, and distance: F(1,52) = 6.37, p = 0.01; Figure 6A). This is indicative of patients exhibiting a reduced replay signature relative to controls, even during epochs enriched for this signal. A similar group ^∗^ session interaction effect was also present when examining change in reactivation separation following learning (pre- to post-learning rest session, interaction effect: F(1,52) = 5.52, p = 0.02) (Wilson and McNaughton, 1994).

Next, to probe the function of spontaneous memory reactivations in “stitching together” associative memories, within the same “high co-activation” epochs we quantified the degree to which two states separated by an intermediate state (e.g., A-[]-C, an *inferred* association) are reactivated preferentially in the presence of their shared intermediate state (e.g., A-B-C) (see Figure 6B for details). The increase in such an “inferential reactivation” signature following learning was augmented in controls relative to patients (group ^∗^ session mixed ANOVA, interaction: F(1,52) = 4.46, p = 0.04; main effects of group: F(1,52) = 0.14, p = 0.71, and session: F(1,52) = 3.16, p = 0.08; Figure 6B), in line with transitive inference impairments reported in patients (Adams et al., 2020; Titone et al., 2004) and the role of hippocampus in supporting this function (Barron et al., 2020; Dusek and Eichenbaum, 1997; Liu et al., 2019).

### Distorted online neural map representations in patients

**Figure 7:**
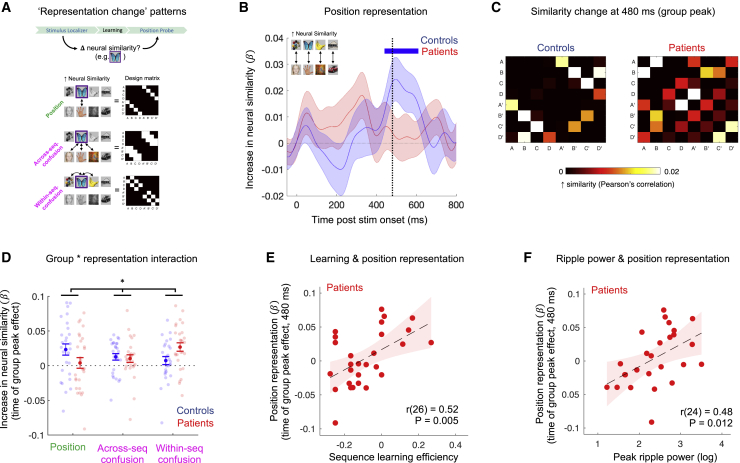
Distorted online neural map representations in patients (A) Hypothesized learning-induced “similarity change” patterns and associated [state × state] design matrices. (B) Effect of emergent (learning-induced) “position” representation at each time bin of the evoked neural response in patients and controls separately (mean ± SEM of subject-level regression weight on the "position" regressor in a multiple regression controlling for “confusion” representations in A). Horizontal blue line denotes cluster P_FWE_ < 0.05 in controls. See Figure S5B for results relating to “confusion” predictors. (C) Mean [state × state] “similarity change” matrices from time point of peak "position" representation in combined sample of patients and controls (480 ms, vertical dashed line in B). See Figure S5C for results relating to “confusion” predictors. (D) Group ^∗^ representation interaction effect (^∗^p < 0.05 interaction, group ∗ representation ANOVA). See Figure S6B for results by medication status. (E) Linear relationship between sequence learning efficiency and peak position representation in patients. See Figure S5D for non-significant effect in controls. (F) Linear relationship between peak replay-conditional ripple power (post-learning rest) and peak position representation in patients. See Figure S5E for non-significant effect in controls. For (B–E), patients: n = 28 (red), controls: n = 27 (blue). For (F), patients: n = 26. Shaded patch in (E and F) designates 95% confidence intervals for line of best fit.

**Figure S5:**
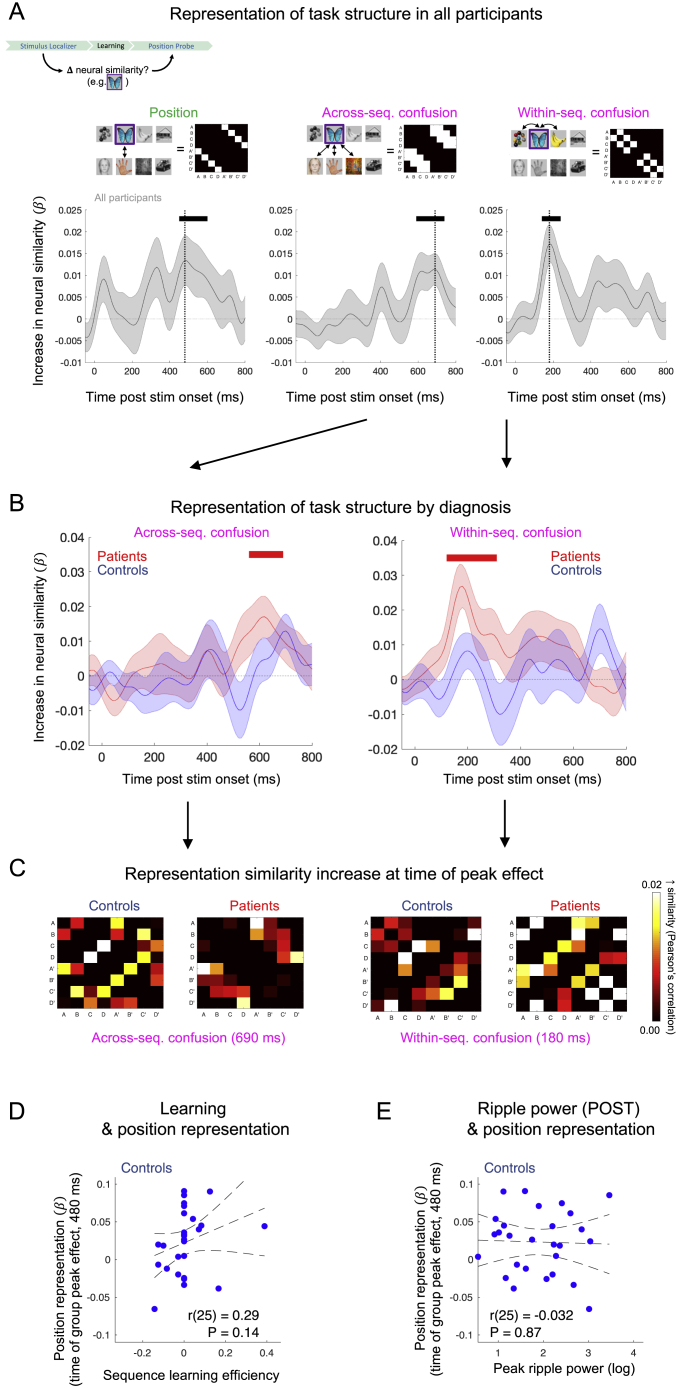
Representational similarity analysis, related to Figure 7 (A) Mean ± SEM representation effects in combined (n = 55) sample of patients and controls. We quantify the change in representational similarity (correlation distance) for each pair of pictures from Stimulus Localizer to Position Probe, and plot the unique variance in the resulting [state x state] similarity change matrix explained by each of three hypothesized representational patterns (binarized design matrices: position representation (Left), across-sequence confusion (Middle) and within-sequence confusion (Right)) for each time point post-stimulus onset. Time windows (clusters) exhibiting positive evidence at P_FWE_ < 0.05 denoted by horizontal bar (cluster-based significant thresholds obtained by shuffling state labels in neural representational dissimilarity matrices). Vertical lines indicate time of peak group mean representation effect (480 ms position representation, 690 ms across-sequence confusion, 180 ms within-sequence confusion). (B) Representation effect corresponding to the two ‘confusion’ predictors, plotted as mean ± SEM in patients (n = 28, red) and controls (n = 27, blue) separately, including group-specific time windows (clusters) exhibiting positive evidence at P_FWE_ < 0.05 (bold horizontal line: patients = red, controls = blue. Patients alone exhibit significant evidence for confusion representations). For position representation, see Figure 7B. (C) Group average ‘similarity change’ matrices from time point exhibiting maximal evidence for representation-specific effect in the combined sample (vertical dashed lines in A: across-sequence confusion: 690 ms, within-sequence confusion: 180 ms) for patients and controls separately. (D) No significant relationship between emergent position representation and sequence learning efficiency in controls (r(25) = 0.29, p = 0.14). See Figure 7E for positive correlation in patients. (E) No significant relationship between emergent position representation and replay-associated peak ripple power (POST learning) in controls (r(25) = 0.03, p = 0.87). See Figure 7F for positive correlation in patients. For D – E error bars represent 95% confidence intervals for line of best fit..

Finally, we asked whether abnormalities in offline reactivation signatures in patients might relate to emergence of a distorted neural representation of task structure following learning, given the proposed function of replay and SWRs in formation and extension of neural map-like representations (Barron et al., 2020; Behrens et al., 2018; Gupta et al., 2010; Joo and Frank, 2018; Liu et al., 2019; Sugden et al., 2020). Our paradigm included structurally identical localizer tasks both before and after Applied Learning (Figures 1C and 7A), allowing us to quantify a learning-induced *change* in neural similarity (correlation distance) of whole-brain evoked response for each pair of pictures, using a Representational Similarity Analysis (RSA) (Deuker et al., 2016; Diedrichsen and Kriegeskorte, 2017). This procedure generates a [state × state] “similarity change” matrix for each time point in the evoked neural response following picture-onset. For each participant, we regressed the matrices from each time point onto a task design matrix comprising predictors encoding a correct abstracted “position” representation (i.e., increased similarity for pictures occupying the same ordinal position in different sequences; Liu et al., 2019; Luyckx et al., 2019), and two predictors capturing plausible alternative (“confusion”) representations (i.e., increased similarity for adjacent pictures, either within or across sequences, shown in Figures 7A and S5A).

**Figure S6:**
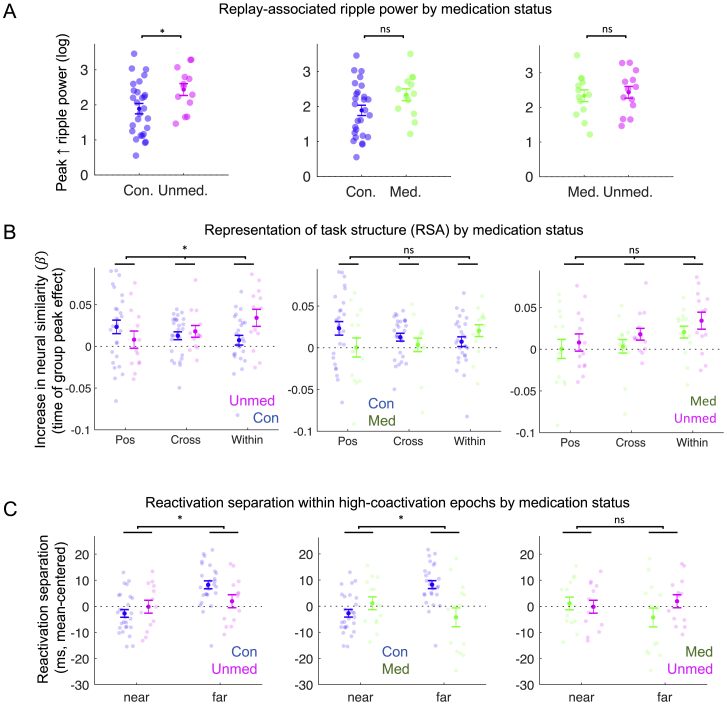
Group differences in neural effects, stratified by medication status, Related to Figures 5, 6, and 7 (A) Replay-associated ripple power in post-learning rest (peak power increase in ripple range, 120 – 150 Hz). (Left) Controls versus unmedicated patients t(38) = −2.24, p = 0.031. (Middle) Controls versus medicated patients t(38) = −1.83, p = 0.075. (Right) Medicated versus unmedicated patients t(24) = −0.41, p = 0.68. Unpaired t test, 2 tailed. Sample: controls n = 27, unmedicated patients n = 13, medicated patients n = 13. See Figure 5C for control versus patient result. (B) Learning-induced increase in representational similarity from Stimulus Localizer to Position Probe, where representation effect is extracted at time of group peak effect after picture onset. (Left) Controls versus unmedicated patients, group ^∗^ representation interaction: F(1, 38) = 3.16, p = 0.048. (Middle) Controls versus medicated patients, group ^∗^ representation interaction: F(1, 40) = 2.30, p = 0.11. (Right) Medicated versus unmedicated patients, group ^∗^ representation interaction: F(1, 26) = 0.07, p = 0.93. Sample: controls n = 27, unmedicated patients n = 13, medicated patients n = 15. See Figure 7D for control versus patient result. (C) Reactivation separation analysis in post-learning rest: temporal separation of structurally adjacent states (‘near’, [A-B]) versus states separated by a single intermediate state (‘far’, [A-C]), within high-coactivation epochs. (Left) Controls versus unmedicated patients, group ^∗^ distance interaction: F(1, 38) = 5.56, p = 0.024. (Middle) Controls versus medicated patients, group ^∗^ distance interaction: F(1, 39) = 12.52, p = 0.001. (Right) Medicated versus unmedicated patients, group ^∗^ distance interaction: F(1, 25) = 1.69, p = 0.21. Sample: controls n = 27, unmedicated patients n = 13, medicated patients n = 14. See Figure 6A for control versus patient result. All effects plotted as mean ± SEM.

Across all participants, we found evidence for the emergence of an abstracted position representation from 450–600 ms after stimulus onset (cluster-level P_FWE_ = 0.032, non-parametric permutation test; peak 480 ms: β = 0.013 ± 0.006, t(54) = 2.37, p = 0.022, uncorrected two-tailed one-sample t test), in addition to “confusion” representations reflecting an increase in neural similarity for adjacent pictures, both within (140–240 ms, P_FWE_ = 0.032; peak 180 ms: β = 0.017 ± 0.004, t(54) = 3.96, p = 0.0002 uncorrected) and across sequences (590–740 ms, P_FWE_ = 0.013; peak 690 ms, β = 0.011 ± 0.004, t(54) = 3.17, p = 0.003 uncorrected; Figure S5A). Notably, the representational patterns differed markedly between groups. Control participants alone exhibited a significant position representation (Figures 7B and 7C), while patients instead exhibited significant “confusion” representations (Figure S5B and S5C). Accordingly, there was a significant interaction effect in a group ^∗^ representation ANOVA, where the effect for each representation was extracted from the time of peak effect across all participants (F(2,106) = 4.03, p = 0.02; Figure 7D; no main effect of group: F(1,53) = 0.03, p = 0.86, or representation: F(2,106) = 0.35, p = 0.71).

Abstracted structural representations are hypothesized to facilitate relational inferences in novel, yet structurally congruent, environments (Behrens et al., 2018; Dragoi and Tonegawa, 2013; Liu et al., 2019; Luyckx et al., 2019). In keeping with this, we found a positive relationship between sequence learning efficiency and the strength of an emergent position representation in patients (r(26) = 0.52, p = 0.005; Figure 7E). Although this correlation was not significant in controls (r(25) = 0.29, p = 0.14; Figure S5D), the linear association between learning efficiency and position representation was significant across all participants in a multiple regression analysis, with no evidence for a significant group difference (representation ∼ group ^∗^ sequence learning efficiency regression, β_learning_ = 0.13 ± 0.045, t(51) = 2.99, p = 0.004; β_group_ = 0.005 ± 0.011, t(51) = 0.48, p = 0.64; β_interaction_ = −0.031 ± 0.090, t(51) = −0.35, p = 0.73). By contrast, “confusion” predictors showed no relationship to sequence learning efficiency (Table S2).

SWRs play a key role in stabilization of neural map-like representations (Barron et al., 2020; Buzsáki, 2015; Fernández-Ruiz et al., 2019; Joo and Frank, 2018; Roux et al., 2017; Sugden et al., 2020), and augmented ripple power in rodent models of schizophrenia may serve to enhance this stabilization in the context of impaired replay (Suh et al., 2013; Zaremba et al., 2017). Consistent with this hypothesis, peak replay-associated ripple power during post-learning rest was associated with the strength of an emergent position representation in patients (r(24) = 0.48, p = 0.012; Figure 7F) but not controls (r(25) = −0.03, p = 0.87; Figure S5E), and multiple regression analysis confirmed that this group difference was significant (representation ∼ group ^∗^ ripple regression, β_interaction_ = −0.035 ± 0.017, t(49) = −2.08, p = 0.042; β_group_ = 0.027 ± 0.012, t(49) = 2.27, p = 0.03. β_ripple_ = 0.016 ± 0.008, t(49) = 1.87, p = 0.068). In a series of control analyses, we find no relationship between ripple power detected using pre-learning rest and emergent position representation, and no relationship between post-learning ripple power and “confusion” representations. We also find no relationship between position representation and post-learning neural replay (Table S2).

### Relationship of behavioral and neural effects to clinical variables

In our patient sample, we found no evidence for a monotonic relationship between any clinical variable and either sequence learning efficiency or (post-learning) peak sequenceness (Table S3). Our reported neural and behavioral group differences are not attributable to medication effects (Figures S1C, S1D, S2C, S2D, S3C, and S6).

## Discussion

The hippocampal abnormalities in our study have a striking similarity to genetic mouse models of schizophrenia that perturb cortical excitation-inhibition balance, which show both increased hippocampal SWR abundance and power during rest (Altimus et al., 2015; Suh et al., 2013; Zaremba et al., 2017), as well as abnormal coordination of place cell reactivations indicative of impaired sequential replay (Suh et al., 2013). Despite such correspondences, the specificity of these hippocampal abnormalities for schizophrenia, or related psychotic disorders, remains an open question. Pathological and hypersynchronous hippocampal discharges (e.g., pathological ripples and interictal spikes) are also a feature of temporal lobe epilepsy (TLE) and relate to cognitive impairment associated with this condition (Buzsáki, 2015; Gelinas et al., 2016). Thus, at the neurophysiological level, TLE and some manifestations of psychotic disorders may be related, and we note that epilepsy (especially TLE) is a significant risk factor for schizophreniform psychosis (Clancy et al., 2014; Radua et al., 2018). Intriguingly, one preclinical model of TLE reports abnormal interictal discharges in hippocampus in the context of relatively normal spontaneous replay (Titiz et al., 2014). We believe that the further characterization of ripple and replay properties in preclinical models of psychosis and TLE, and how they relate to symptomatic manifestations, provides an exciting avenue for future work.

Our findings of impaired neural replay in schizophrenia embrace more general neurobiological theories of psychosis. For example, hippocampal hyperexcitability and excitation-inhibition imbalance accounts (Grace, 2016; Krystal et al., 2017; McHugo et al., 2019) are proposed to manifest in abnormal temporal dynamics of place cell reactivations (i.e., replay) during rest (Buzsáki, 2015; Krystal et al., 2017; Suh et al., 2013). Similarly, abnormal replay is predicted by glutamate models (McCutcheon et al., 2020a), given a necessary role of *N*-methyl-D-aspartate (NMDA) receptors in stabilizing hippocampal maps (Dupret et al., 2010) and encoding new sequential episodes (Foster, 2017; Silva et al., 2015) and HEC-dependent relational schemas (Dragoi and Tonegawa, 2013). Finally, given a dominant dopamine hypothesis of schizophrenia has, to date, focused on overactivity in a meso-striatal circuit (Howes and Kapur, 2009; Maia and Frank, 2017; Schmack et al., 2021), it is of interest that abnormal dopamine signaling has been proposed to arise secondary to hippocampal hyperexcitability (Grace, 2016; Suh et al., 2013).

The HEC represents the inferred relational structure of non-spatial domains (Behrens et al., 2018; Bellmund et al., 2018; Constantinescu et al., 2016; Garvert et al., 2017; Luyckx et al., 2019; O’Keefe and Nadel, 1978) and supports a rapid assimilation of new experiences into related cognitive schemas (maps) (Dragoi and Tonegawa, 2013; Dusek and Eichenbaum, 1997; Gupta et al., 2010; Liu et al., 2019; Sun et al., 2020; Whittington et al., 2020). Offline replay and associated SWRs play a fundamental role in memory consolidation and stabilization of these map-like neural representations (Barron et al., 2020; Buzsáki, 2015; Fernández-Ruiz et al., 2019; Joo and Frank, 2018; Lewis et al., 2018; Roux et al., 2017; Sugden et al., 2020). Our findings add to this literature. First, participants who were most efficient in acquiring sequence knowledge showed the strongest signatures for subsequent online structural representations (as measured with RSA) and offline sequential replay (as measured with TDLM), despite the fact that participants in both groups were at ceiling-level knowledge immediately prior to rest. An intriguing yet tentative possibility is that these post-learning neural signatures covary with differences in a neural representation of abstracted task structure, present during learning itself (Liu et al., 2019), and which facilitates rapid assimilation of new experiences into learned HEC-dependent schemas. Second, replay-associated ripple power predicted an emergence of a post-learning neural representation of task structure in patients, in line with a suggestion that augmented SWR power in the context of pathologically impaired replay reflects a compensatory mechanism (Zaremba et al., 2017).

Given the importance of HEC in representing the inferred relational structure of the world, and the role of offline reactivations in maintaining and extending this representation, abnormalities in hippocampal replay might provide for a neurocomputational understanding of schizophrenia. Hippocampal replay has been proposed as relevant for understanding cognitive manifestations related to model-based decision making (Dolan and Dayan, 2013; Liu et al., 2021b; Morris et al., 2018) and inferential reasoning (Adams et al., 2020; Titone et al., 2004), including symptomatic manifestations that stem from inferences about unobserved relationships between entities (e.g., incorrect inferences about other people, as in paranoia). This can be distinguished from impairments in (model-free) association and reward learning following direct experience (i.e., trial and error), which have received considerable research attention (Maia and Frank, 2017) but which do not require a relational cognitive map of the task environment.

Finally, there is a tight coupling between hippocampal SWRs and whole-brain activity in default mode network (DMN) brain regions at rest, including medial temporal lobe (Higgins et al., 2021; Kaplan et al., 2016). Neuroimaging studies in patients with early psychosis report abnormal DMN functional connectivity (Whitfield-Gabrieli et al., 2009) and hippocampal hypermetabolism (McHugo et al., 2019; Schobel et al., 2013) at rest. There is suggestive evidence the latter may relate to high-frequency (ripple) oscillations in hippocampus (Bergel et al., 2020). These findings are intriguing, given the hypothesized role of offline hippocampal/DMN activity in imaginative (compositional) cognition (Barron et al., 2020, 2013; Buckner, 2010; Zeidman and Maguire, 2016), which we and others speculate may play a role in pathologies of belief seen in psychosis (Buckner, 2010; Lewis et al., 2018; Suh et al., 2013).

### Limitations of the study

One concern is a possibility that reported group differences might be driven by differential contributions of other factors such as motivation or attention. We consider this unlikely given that patient and control participants were tightly matched across numerous cognitive and demographic variables, with equivalent performance on measures of task engagement. Furthermore, the replay deficit was expressed under resting as opposed to task conditions. We also highlight equivalent neural state decodability across the scanning session. Moreover, neural and behavioral effects did not correlate with variables known to covary with indices of structural gray matter loss in schizophrenia, such as age, disease chronicity, age at symptom onset, or antipsychotic use (Ho et al., 2011; Hulshoff Pol and Kahn, 2008; Torres et al., 2016).

Second, although we present evidence for an experience-induced change in replay strength related to learning, we acknowledge replay does not relate clearly to subjectively reported symptoms. We propose that a replay deficit may reflect a trait-level abnormality, with a complex relationship to time-varying and context-sensitive symptom-level features. Future studies, especially using causal replay manipulations, are required to test this hypothesis, in addition to related hypotheses regarding the role of replay in cognition.

### Conclusions

In summary, we present converging evidence supporting a neurobiological model of schizophrenia whereby offline replay and ripple abnormalities are associated with a distorted representation of the inferred relational structure of the environment. Our findings open future avenues of investigation that can address how spontaneous neural state reactivations might relate to symptom expression and potential therapeutic avenues, as well as how replay might be implicated in other psychiatric conditions.

## STAR★Methods

### Key resources table

**Table:** 

REAGENT or RESOURCE	SOURCE	IDENTIFIER
**Deposited Data**		

MEG data	This paper	N/A

**Software and Algorithms**		

MATLAB software used for all quantitative analysis	MATLAB	https://www.mathworks.com/products/matlab.html
OHBA Software Library (OSL) for MEG pre-processing and beamforming (MATLAB scripts)	N/A	https://ohba-analysis.github.io/osl-docs/
Methods & simulation code to measure sequential replay (MATLAB scripts). (see Figure 3)	This paper	https://github.com/YunzheLiu/TDLM
Influence of background alpha on sequenceness simulation code (MATLAB scripts) (see Figure S4)	This paper	https://github.com/matthewnour/TDLM_alpha_simulation/

**Other**		

Human healthy participants recruited from local area, and patient participants recruited from local community NHS clinics. (see Table S1)	This paper	N/A
Neural recordings and amplifier	Whole Brain CTF MEG 275 System	https://www.ctf.com/

### Resource availability

#### Lead contact

Further information and requests for resources and reagents should be directed to and will be fulfilled by the Lead Contact, Matthew Nour (matthew.nour.18@ucl.ac.uk).

#### Materials availability

This study did not generate new unique reagents.

#### Data and code availability

Summary data used to generate the findings of this study will be available upon request to the Lead Contact. Individual participant MEG data is available subject to participant consent.

TDLM MATLAB Code available at: https://github.com/YunzheLiu/TDLM & https://github.com/matthewnour/TDLM_alpha_simulation/. Additional custom computer code will be made available upon request to the Lead Contact.

### Experimental model and subject details

#### Participants and assessment

The study was approved by the London Westminster NHS Research Ethics Committee (15/LO/1361). All participants provided written informed consent and were compensated for their time. We recruited 31 patients (6 female, 25 male) with schizophrenia (assessed with the Structured Clinical Interview for DSM-IV-TR, Axis I Disorders, SCID-I (First et al., 1995)) from London community psychosis NHS clinics, and 29 healthy volunteers (6 female, 23 male), from the same geographical area through online advertisements. Groups were matched for age, gender, IQ and educational attainment. General exclusion criteria were anticonvulsant or benzodiazepine medication, age > 45 years, poor vision limiting performance and not having been educated in English. Healthy volunteers were not taking neurological or psychiatric medication, had no history of neurological or psychiatric disorder (assessed by SCID-I (First et al., 1995)), and no family history of psychosis. Patients had no neurological or psychiatric comorbidity. The final sample comprised 28 patients (3 patients declined MEG after training, secondary to paranoia) and 27 healthy volunteers (2 excluded for behavioral accuracy score < 3.5 SD from group median during MEG). 13 patients were not taking D2/3 antagonist medication (1 medication-naive). We assessed psychiatric symptoms with the Positive and Negative Syndrome Scale (PANSS) scale (Kay et al., 1987), Montgomery Åsberg Depression Rating Scale (MADRS) (Williams and Kobak, 2008), and General Assessment of Function (GAF) (American Psychiatric Association, 2013). We administered brief measures of IQ (the Wechsler Test of Adult Reading, WTAR (Wechsler, 2001)) and working memory (mean of forward and backward Digit Span). Cognitive and clinical assessments were conducted following the behavioral training session (visit 1), prior to MEG. See Table S1 for final sample demographic and clinical / cognitive scores.

### Method details

#### Applied Learning task and MEG sessions

Participants performed a validated task during MEG, where they inferred how 8 distinct task pictures were embedded within two separate ‘structural sequences’ ([A→B→C→D] & [A’→B’→C’→D’]), without ever being shown either complete sequence order (Liu et al., 2019). Instead, participants viewed scrambled ‘visual sequences’ containing pictures from both ‘structural sequences’ (Figure 1A). During pre-scan training (visit 1) participants were explicitly shown how 8 training pictures embedded within 3 ‘visual sequences’ could be mapped onto 2 correct ‘structural sequences’. Specifically, they were informed that in each ‘visual sequence’ (e.g., [C→D→C’→D’]) only the first and last transitions correspond to correct ‘structural relationships’ (e.g., [C→D & C’→D’] are ‘structural’ transitions, but [D→C’] is a ‘visual-only’ transition), and their understanding was checked subsequently by asking them to explain this ‘unscrambling rule’. Once this understanding was established, participants completed 3 Structure Learning sessions in which they practiced how to infer the correct structural relationships between the 8 training pictures; learning not only how the specific training pictures were associated, but also encoding a stimulus-independent mapping from ‘visual’ to ‘structural’ sequences (i.e., the ‘unscrambling rule’, an abstracted task schema/cognitive map (Behrens et al., 2018)). During MEG (visit 2) participants completed 3 similar Applied Learning sessions, with an entirely new picture set of 8 pictures, thus applying the encoded task schema to novel stimuli (se Figure 1). The use of a novel picture set for MEG ensures that neural replay signatures measured during MEG cannot be attributed to perceptual biases introduced at training (Liu et al., 2021a, 2019).

In each learning session (both Structure Learning on visit 1, and Applied Learning on visit 2) participants were presented with a set of 3 unique ‘visual sequences’ (e.g., [C→D→C’→D’], [B’→C’→B→C], & [A→B→A’→B’]), and this set was itself repeated 4 times (i.e., 12 sequence presentations) (Figure 1B). In each ‘visual sequence’ presentation the 4 pictures were presented sequentially from left to right, concurrent with a single word text descriptor at the bottom of the screen (each picture 175^∗^175 pixels, presented for 1.5 s, with an inter-stimulus interval of 300 ms separating the first and last pair of pictures, and 1100 ms separating the second pair of pictures. Inter-sequence interval 3 s) (Liu et al., 2019). The structural embedding of each picture within ‘visual’ (and, by extension, ‘structural’) sequences was counterbalanced between subjects.

Following each Applied Learning session, we assessed knowledge of structural sequences (i.e., the picture→state embeddings) with a 12-question quiz. For each question a single target picture (e.g., B) was presented on the center of the screen for 5 s, followed by the appearance of two probe pictures (e.g., A and D) at the bottom left and right of the screen (Figure 2). Participants indicated which of the two probe pictures *comes later* than the target picture in the ‘structural sequence’ with a button press, with no time restrictions or feedback. Laterality of the correct/incorrect pictures was randomly selected on each trial. To assess the granularity of participants’ knowledge of structural sequences, each quiz contained two question types: (1) questions that relied on knowledge of picture order within a sequence (within-sequence questions), and (2) questions that could be answered without knowledge of picture order (cross-sequence questions, see Figure 2 for further details).

During MEG, prior to Applied Learning, participants completed a 5 min rest session with eyes open (pre-learning rest, at the start of the scanning session), which acted as a subject-specific baseline condition for our sequenceness analysis (i.e., controlling for between-subject variance in statistical (e.g., oscillatory) properties of rest MEG data) (Genzel et al., 2020). This was followed by a Stimulus Localizer task, which provided training data for the stimulus decoders required for sequenceness analysis (Figures 1C and 3). On each trial of the Stimulus Localizer participants were presented a task picture on the center of the screen (320^∗^320 pixels, 1 s), followed immediately by presentation of a single-word text descriptor, and indicated whether the text matched (50% trials) or did not match (50% trials) the preceding picture with a button press (2 s response window, no feedback, laterality of yes/no response counterbalanced between subjects). Each picture was shown many times (mean 51.0, SD 3.48) in a randomized order. The inter-trial interval was drawn uniformly from 700 – 1300 ms on each trial. Following Applied Learning, participants completed a second 5 min rest session with eyes open (post-learning rest), which ended with a 4^th^ (post-rest) knowledge quiz to assess knowledge retention. To incentivize learning, participants then completed a further task session where they learned that pictures of one sequence were associated with monetary reward, and could use this knowledge to increase their compensation, as in our prior study (Liu et al., 2019). At the end of the scan session participants completed a Position Probe task (Figure 1C). This differed from the Stimulus Localizer task in one respect alone. Here, each picture was followed by a single *number* (1, 2, 3 or 4), and participants now indicated whether this number matched the *position* of the preceding picture with a button press (2 s response window, no feedback, chance accuracy 50%, laterality of yes/no response counterbalanced between subjects). Each stimulus was presented a mean of 35.9 [6.5] times for each subject, in randomized order. By comparing neural data from Stimulus Localizer to Position Probe, we can assess learning-induced changes in the representational similarity between picture pairs in the visually evoked response.

The task was implemented in MATLAB (MathWorks) using Cogent (Wellcome Trust Centre for Neuroimaging, University College London) v 1.30.

### Quantification and statistical analysis

#### MEG acquisition and preprocessing

MEG was recorded continuously at 1200 samples/second using a whole-head 275-channel axial gradiometer system (CTF Omega, VSM MedTech), while participants sat upright (3 sensors not recorded due to excessive noise in routine testing). The task was projected onto a screen suspended in front of participants, and participants responded using two buttons (L/R) of a MEG-compatible button box (Current Designs) held in the right hand. Preprocessing was conducted separately for each session, identical to our previous study (Liu et al., 2019). Sensor data were high-pass filtered at 0.5 Hz using a first-order IIR filter to remove slow-drifts. Data were then down sampled (including anti-aliasing low-pass filter) to 100 Hz (sequenceness and representational similarity analysis) and 400 Hz (time-frequency analysis), and excessively noisy segments and sensors automatically removed before independent component analysis (ICA). ICA (FastICA, http://research.ics.aalto.fi/ica/fastica) was used to decompose the sensor data for each session into 150 temporally independent components and associated sensor topographies. Artifact components (e.g., eye blink and mains interference) were classified by automated inspection of the spatial topography, time course, kurtosis of the time course and frequency spectrum for all components (Liu et al., 2019). Artifacts were rejected by subtracting them out of the data. Epoched MEG data from Stimulus Localizer and Position Probe trials was baseline-corrected (mean of 100 ms pre-stimulus baseline subtracted from data) before analysis. All analyses were performed on the filtered, cleaned MEG signal from each sensor, in units of femtotesla. All MEG analyses (except source localization) were performed at whole-brain sensor level.

There was no significant difference between controls and patients in the mean number of rejected MEG channels over all sessions (mean controls = 10.44 (SD 10.50), patients = 14.64 (9.50). t(53) = −1.56, p = 0.13, two-tailed unpaired t test), nor the proportion of bad samples excluded in the examined rest sessions (pre-learning rest: controls = 0.10 (0.08), patients = 0.11 (0.10). t(53) = −0.24, p = 0.81. post-learning rest: controls = 0.11 (0.09), patients = 0.11 (0.10). t(53) = −0.07, p = 0.94, two-tailed unpaired t test). These group comparisons remain non-significant (p > 0.05) when restricting the patient sample to those currently on or off D2/3R antagonist medication.

#### Stimulus decoding

Sequenceness analysis relies on the ability to quantify evidence for transient spontaneous neural reactivations of task stimuli from MEG sensor patterns during rest (Figure 3). For each stimulus (k ∈{1:8}) we trained a separate one-versus-rest lasso-regularized logistic regression model using data from Stimulus Localizer, at each 10 ms time bin from −100 ms to +1000 ms post-stimulus onset. Each model, k, discriminated between sensor patterns pertaining to stimulus k (positive examples) compared to all other stimuli (∼k) plus an equivalent amount of ‘null’ data from the inter-trial interval (negative examples). Inclusion of null data reduces the spatial correlation between decoders, enabling all decoders to report low probabilities simultaneously in rest data (Liu et al., 2021a, 2019). A stimulus- and time-specific model, k, thus consisted of a single vector of length n+1 (n sensors [max. 272], plus intercept). L1 regularization was used to encourage sparsity and enhance sensitivity for sequence detection (Liu et al., 2021a). To ensure the results were not overfit to the regularization parameter, λ, we fixed λ = 0.006 (this is the modal value that optimized detection of sequenceness in leave-one-out cross-validation in our previous study in an independent sample (Kurth-Nelson et al., 2016). A similar cross-validation procedure in our own data yields similar parameters and equivalent statistical results).

To quantify *within-session* decoding accuracy, regression models were trained in leave-one-out cross-validation (train and test examples from Stimulus Localizer). Prediction accuracy on each validation loop was quantified as the proportion of test trials (n = 8, one for each stimulus class) where the decoder reporting the highest probability corresponded to the trial label, with overall accuracy defined as the mean of this estimate over all validation loops (Figures 4A and S2C) (Kurth-Nelson et al., 2016; Liu et al., 2019; Wimmer et al., 2020). To quantify *cross-session* accuracy we applied the decoders trained on Stimulus Localizer data (start of scan) to each trial of the Position Probe (end of scan), and estimated accuracy identically (Figures S2D and S2E). Both analyses yielded maximum decoding accuracy for decoders trained (and tested) at 180 ms post-stimulus onset (mean accuracy over all participants) (Figures 4A, S2C, and S2D). Consequently, decoders from this time bin were used for sequenceness analysis (Kurth-Nelson et al., 2016; Liu et al., 2019; Wimmer et al., 2020). We confirmed that decoding accuracy was significantly greater than chance (expected chance decoding accuracy 1/8 = 12.5%) using nonparametric tests. Specifically, we permuted the labels of test trials 500 times per participant, and for each permutation identified the maximal mean accuracy over participants from −100 to +1000 ms post-stimulus onset (thus controlling for multiple tests over time), to generate an empirical null distribution. Group mean accuracy in the unpermuted data was deemed significant at peak-level P_FWE_ < 0.05 if it exceeded the 95^th^ percentile of this empirical null distribution.

#### Sequenceness

The sequenceness analysis pipeline is illustrated in Figure 3. We first applied trained decoders (from the peak accuracy time bin) to MEG data from each time point of pre- and post-learning rest sessions, to generate a [time x state] reactivation probability matrix for each session (Liu et al., 2019; Wimmer et al., 2020). We then used the Temporally Delayed Linear Modeling (TDLM) framework to quantify evidence for sequential reactivations consistent with the inferred task transition structure (Liu et al., 2021a, 2019; Wimmer et al., 2020).

TDLM is a multiple linear regression approach that quantifies the degree to which a lagged (i.e., past) reactivation time course of state i, (X(Δt)_i_, t indicates lag time) can predict the reactivation time-course of state j, (X_j_) (Liu et al., 2021a, 2019; Wimmer et al., 2020). We first performed a separate (first-stage) family of multiple regressions using each state’s (j ∈ {1:8}) reactivation time course as a dependent variable, and the historical (i.e., time-lagged) reactivation time courses of all states (i ∈ {1:8}) as predictor variables:

1 $${\text{X}}_{\text{j}}=\sum_{\text{i}=1}^{8}\text{X}{\left(\Delta \text{t}\right)}_{\text{i}}\text{∗}\beta {\left(\Delta \text{t}\right)}_{\text{i,j}}+\text{C}\;$$

The predictor (design) matrix from a single model contained a separate predictor for the reactivation time courses of all states (i ∈ {1:8}), lagged by Δt ∈ {10 ms, 20 ms, …, 90 ms}, plus the reactivation time course of all states lagged by Δt+α, where α = [100 ms, 200 ms, … ], to capture autocorrelations in state time courses at a canonical alpha frequency, which predominates in human brain activity at rest (Liu et al., 2021a, 2019) (see Figure S4), in addition to a constant term, C. We repeat the regression in Equation 1 for each j ∈ {1:8} and Δt ∈ {10, 20, 30, …, 90 ms}, and use ordinary least-squares regression to obtain β.

The regression coefficients from Equation 1 quantify the evidence for each empirical state→state reactivation pattern at a specific lag. Δt. For example, β(Δt)_i,j_ is the coefficient capturing the unique variance in X_j_ explained by X(Δt)_i_. All such first-level coefficients are represented in a lag-specific [8 × 8] empirical transition matrix B.

In a second-level regression, we then quantified the evidence that the empirical (lag-specific) transition matrix, B, is predicted by the underlying task transition structure (i.e., ‘structural sequences’),

2 $$\text{B}=\sum_{\text{r}=1}^{4}{\text{Z}}_{\text{r}}\text{∗}{\text{T}}_{\text{r}}$$

where B is the [state x state] empirical transition matrix, T_r_ is the [state x state] predictor transition matrix (for regressor r), and Z_r_ is the scalar regression coefficient quantifying the evidence that the hypothesized transitions, T_r_ predict the empirical transitions, B. We consider 4 predictor matrices, T (i.e., r ∈ {1:4}): (1) ‘structural sequence’ transitions in the *fwd* direction (transitions corresponding to [A→B→C→D] and [A’→B’→C’→D’] in T_1_ are set to 1, all other transitions set to 0), (2) ‘structural sequence’ transitions in the *bwd* direction ([D→C→B→A] and [D’→C’→B’→A’], i.e., T_2_ is the transpose of T_1_), (3) self-transitions ([8 × 8] identity matrix), and (4) a constant matrix.

In this study, sequenceness is defined from the contrast between evidence for sequential replay of task structure in the forward ([A→B→C→D]) versus backward ([D→C→B→A]) direction (i.e., Z_1_ - Z_2_), thus removing between-subject variance in sequential replay per se (which may arise secondary to task engagement and measurement sensitivity (Liu et al., 2021a)). Positive sequenceness values indicate replay in a predominantly forward direction, which has been previously shown to emerge in an awake rest session following learning in similar MEG paradigm (Liu et al., 2019). Of note, in our study and previous studies (Liu et al., 2019) participants are explicitly encouraged to conceive of the inferred structures as ordinal linear sequences progressing in a (forward) direction from 1^st^ → 4^th^, and are prompted to think in this direction-specific manner in the post-learning quiz session (Figure 2). We estimate sequenceness at each state→state transition lag (10 – 600 ms, in 10 ms bins), by repeating the regression of Equation 2 separately for the empirical transition matrix, B, corresponding to each Δt, and report this effect SD-scaled over lags within participants. Sequenceness at shorter lags indicates greater time compression (Kurth-Nelson et al., 2016; Liu et al., 2019; Wimmer et al., 2020).

For statistical testing we used nonparametric tests at the second-level regression of TDLM, shuffling the rows and columns of T_1_ (*forward* predictor matrix), defining T_2_ (*backward* predictor matrix) as its transpose. For each of 1000 permutations we calculated the peak absolute mean sequenceness over participants and across lags (thus controlling for multiple comparisons across lag), to generate an empirical null distribution. The group mean sequenceness effect in the unpermuted data was deemed significant (at peak-level P_FWE_ < 0.05) if its absolute magnitude exceeded the 95^th^ percentile of this empirical null distribution (Liu et al., 2021a, 2019).

#### Identifying replay onsets and time-frequency analysis

Having found maximal evidence for replayed transitions at 40 ms lag in the combined sample of all participants, we next identified time points during rest where strong reactivation of one stimulus (e.g., A) was followed by strong reactivation of a structurally-adjacent stimulus (e.g., B), 40 ms later (Liu et al., 2019). We first generated a matrix Orig as

3 Orig=X∗T

where X is the [time x state] reactivation matrix, and T is the task transition matrix. The transition matrix T defines the mapping between the task state corresponding to column i in X, and column i in Orig (specifically, column i in Orig is the reactivation time course of the state that ‘precedes’ state i in T). We then shifted each column of X by Δt=40 ms, to generate another matrix Proj,

4 Proj=X(Δt)

where row i of Proj correspond to row i+40 ms of X. Multiplying Proj and Orig elementwise, and summing over the columns of the resulting matrix, therefore yields a [time x 1] vector, R, where each element, t, corresponds to the evidence for a two-state replay with 40 ms lag, starting from any task state at time t.

5 $${\text{R}}_{\text{t}}=\sum_{\text{i}=1}^{8}{\text{Orig}}_{\text{ti}}\text{∗}{\text{Proj}}_{\text{ti}}$$

We identified putative replay event onsets as time points in R > 95^th^ percentile of the subject- and session-specific distribution, and preceded by 200 ms pre-onset baseline exhibiting summed reactivation evidence < 90^th^ percentile at each time point (as per Reactivation Analysis, below) (Liu et al., 2019; Wimmer et al., 2020). We then epoched the rest data surrounding each event and computed a frequency decomposition (wavelet transformation) in the window −100 to +150 ms with respect to replay onset, for each (non-artifactual) sensor and event. Averaging this estimate over sensors and events resulted in a [time x frequency] matrix for each participant, capturing the typical spectrally-resolved power change at replay onset. For each participant, we defined a separate [time x frequency] matrix using forward and backward task transition matrices (i.e., replay of structurally-adjacent states), and used the average of these matrices for subsequent analyses. Our primary analysis tested whether there was evidence for a power increase in the ripple-band at replay onset (0 ms ± 10 ms), compared to a pre-onset baseline (−100 to −10 ms from onset), where the ‘ripple-band’ was defined as 120 – 150 Hz, informed by our previous study (Liu et al., 2019). Having established this effect, we examined whether groups differed in peak ripple power increase over the entire length-2 replay window (0–50 ms from onset, ± 10 ms). For all analyses, individual participant [time x frequency] matrices were SD-scaled within-frequency and across time prior to group level inference.

We conducted an additional exploratory analysis of the temporal dynamics of ripple power fluctuations during rest. For each subject and rest session, we derived the instantaneous ripple power for each sample (Hilbert transform on MEG data, sampled at 400 Hz, following bandpass filtering to 120 – 150 Hz, using FIR filter design using window method). Power was quantified as the squared absolute value of the analytic signal, and averaged over all (non-artifactual) MEG sensors. We defined a discrete ripple ‘event’ as any sample exhibiting ripple power exceeding the median + 2 standard deviations of the subject- and session-specific distribution (we obtain similar results when using event-defining thresholds from 2 – 6 SD) (Gillespie et al., 2021; McNamara et al., 2014; van de Ven et al., 2016). Event rate, interval times, and lifetimes were then calculated for each subject and rest session, in a manner that takes into account the presence of bad samples (Higgins et al., 2021).

#### MEG source reconstruction

We next identified neural sources correlating with increased ripple power at identified replay onset times (i.e., time points during rest where strong reactivation of one stimulus was followed by strong reactivation of a structurally-adjacent stimulus, with 40 ms lag. Event identification and epoching outlined above). Forward models were generated on the basis of a single shell using superposition of basis functions that approximately corresponded to the plane tangential to the MEG sensor array. Linearly constrained minimum variance beamforming (Van Veen et al., 1997) was used to reconstruct the epoched MEG data to a grid in MNI space (grid step = 5 mm). The sensor covariance matrix for beamforming was estimated using data restricted to 120 - 150 Hz (Liu et al., 2019). All non-artifactual replay epochs were baseline corrected at source level (baseline defined as mean power −100 ms to −10 ms with respect to replay onset). The second level design matrix included separate regressors for main effect of group, subject-specific mean ripple (120 – 150 Hz) power increase at replay onset, and the group ^∗^ ripple interaction. We report whole-brain results for voxels predicted by subject-specific ripple power at onset (0 ± 2.5 ms), and use non-parametric permutation tests on this volume to identify clusters significant at P_FWE_ < 0.05 (whole-brain corrected, cluster-defining threshold t = 3, 5000 permutations).

#### Reactivation analyses

To further investigate the temporal structure of reactivations within rest periods of high spontaneous coactivation (Barron et al., 2020; Buzsáki, 2015; Ji and Wilson, 2007; Wilson and McNaughton, 1994), we first estimated summed reactivation evidence over all decoders at each 10 ms time bin (coactivation evidence). We identified bins where this estimate exceeded the session-specific 95^th^ percentile, and was preceded by 200 ms baseline of coactivation evidence < 90^th^ percentile (analogous to identifying replay onsets, above), and defined a ‘high coactivation epoch’ as ± 200 ms centered on this time point.

For ‘reactivation separation’ analysis, for each participant we first calculated the median time separating suprathreshold reactivations of each pair of decoders within each epoch (which we term the ‘reactivation separation’), and then asked whether the median reactivation separation over all epochs was shorter for structurally-adjacent decoder pairs (‘close’, e.g., [A & B]) compared to state pairs separated by a single intermediate state (‘far’, e.g., [A & C]) (Suh et al., 2013) (threshold = 95^th^ percentile session- and decoder-specific reactivation distribution). In epochs where only one decoder in a pair had a suprathreshold event, we set the reactivation separation equal to the epoch window (we obtain equivalent statistical results when omitting these single-reactivation epochs from analysis). By defining epochs using summed simultaneous reactivations (not lagged reactivations, as above), this analysis is unbiased with respect to detecting reactivation separation patterns that mirror the task transition structure. See Figure 6A for schematic.

For ‘inferential reactivation’ analysis we again focused on reactivation patterns within high coactivation epochs (defined above). We aimed to quantify the degree to which coactivation of an unobserved (2-step) association requiring transitive inference (e.g., [A & C]) was seen in the presence of coactivations of its constituent observed associations (e.g., [A & B] and [B & C]), capturing the putative function of spontaneous memory reactivation in SWRs in ‘stitching together’ associative memories (Barron et al., 2020). For each unique pair of decoders, we first scored each high coactivation epoch according to whether it contained suprathreshold reactivation of both decoders. For each unique pair we then quantified the Sørensen–Dice similarity (i.e., overlap) (Dice, 1945) between the set of epochs containing the paired reactivation (e.g., [A & C]), and the set containing the original pair and each of the 6 remaining other states, separately (i.e., [A & C & B], [A & C & D], [A & C & A’], [A & C & B’], [A & C & C’], and [A & C & D’]). This quantifies the degree to which each paired reactivation ([A & C]) is seen in the presence of a ‘shared’ (third) state (e.g., B, D, A’, B’, C’ or D’), controlling for between-state differences in absolute reactivation. We defined the ‘inferential reactivation’ effect as the mean Sørensen–Dice similarity (Dice, 1945) estimates for ‘structural triplets’ (i.e., where the ‘shared’ state is structurally intermediate to the initial pair, e.g., [A & C & B]), divided (normalized) by the mean similarity estimate for all unique triplets (such that effects > 1 indicate a positive ‘inferential reactivation’ for structural triplets). See Figure 6B for schematic.

#### Representational similarity

We used a Representational Similarity Analysis (RSA) (Diedrichsen and Kriegeskorte, 2017) to investigate the emergence of a representation of task structure from the Stimulus Localizer (pre-learning) to Position Probe (post-learning) sessions. First, for each session we z-scored the pre-processed MEG data over all trials, for each sensor and time point post stimulus-onset (t, −100 to +800 ms). We then regressed the [trial x 1] neural data, Y(s)_t_ (from time point, t, and sensor, s) onto a session design matrix, X, denoting the stimulus label of each trial (dummy coded) (Luyckx et al., 2019),

6 $$\text{Y}{\left(\text{s}\right)}_{\text{t}}=\text{X∗}\beta {\left(\text{s}\right)}_{\text{t}}$$

and used the resulting [stimulus x 1] vector of regression weights, β(s)_t_, as an estimate of the unique activation for each stimulus, in sensor s at time point t. Repeating this procedure over all sensors yielded a [sensor x stimulus] matrix at each time point, which was spatially pre-whitened (to increase reliability of inference) (Diedrichsen and Kriegeskorte, 2017) prior to calculating the Pearson correlation distance between the sensor patterns for each pair of pictures (columns). This generated a symmetrical [8 × 8] Representational Dissimilarity Matrix (RDM) at each time point (Deuker et al., 2016). We conducted this procedure identically in both Stimulus Localizer (SL) and Position Probe (PP), enabling us to calculate the learning-induced increase in representational similarity (similarity change) at each time point, ΔS_t_, as

7 $$\Delta {\text{S}}_{\text{t}}=\text{ RDM}{\left(\text{SL}\right)}_{\text{t}}-\text{ RDM}{\left(\text{PP}\right)}_{\text{t}}$$

where entry s_ij_ of ΔS_t_ quantifies the post-learning similarity increase between evoked signals for stimuli i and j, at time t (Deuker et al., 2016). Finally, we used a second multiple regression to quantify the variance in ΔS_t_ that was uniquely explained by an abstracted representation of ordinal position, controlling for two hypothesized ‘confusion’ representations, for each participant at each time point (i.e., the multiple regression included 3 predictor variables corresponding to each of the hypothesized representational patterns in Figures 7A and S5A, plus an intercept). ΔS_t_ was smoothed over time with a 30 ms Gaussian kernel prior to this second regression (Luyckx et al., 2019).

We used nonparametric tests to identify time windows (clusters) with significant positive evidence for each predictor, correcting for multiple comparisons over time. Specifically, for a given predictor (e.g., position), at each time point post-picture onset we conducted a separate one-sample t test on the representational effect (regression weights) over participants, to obtain the evidence for an effect > 0 (i.e., t-value). We computed the sum of t-values within each continuous stretch of time points exhibiting a positive effect at p < 0.05. We then repeated this procedure for 1000 permutations, on each occasion shuffling the rows and columns (stimulus labels) of ΔS_t_ prior to the second regression (shuffled order consistent across time within each permutation to preserve temporal smoothness in visually evoked neural data). We then extracted the maximal sum-of-t value for the group mean effect in each permutation, identically to in the unpermuted data, to generate an empirical null distribution for the predictor in question. A suprathreshold cluster in the unpermuted data was deemed significant at P_FWE_ < 0.05 if its sum-of-t values exceeded the 95^th^ percentile of this empirical null distribution (Eldar et al., 2018).

#### Statistical analysis of group differences and software

For each MEG analysis, participants with effect sizes > 3.5 SD ± group median were excluded as outliers (1 medicated patient for sequenceness analysis, and 2 medicated patients for replay-conditional time-frequency analysis). For group differences or correlation analyses involving neural effects, we first extracted the neural effect for each subject from a temporal region of interest defined using the mean effect over all participants. For sequenceness this corresponded to the transition lags with post-learning effects surpassing significance at P_peak_ < 0.05 (FWE-corrected across lags). For RSA this corresponded to the time point exhibiting maximal learning-induced representational change for each predictor (although we obtain similar results when using the mean estimate within the cluster showing a significant effect in the combined sample). Prior to testing for group differences in behavioral/neural effects using parametric tests (e.g., unpaired t test, mixed ANOVA), we first conducted a formal test that the effect in question was sampled from a population with a normal distribution (Shapiro Wilk test), and used non-parametric equivalent tests where this null hypothesis was rejected at p < 0.05. For between-subjects multiple regression analyses, ‘group’ was effects coded (patients = −0.5, controls = +0.5). Continuous predictor variables were not mean centered in cases where the variable was already a contrast of two effects (i.e., sequenceness, sequence learning efficiency), but were mean-centered otherwise (i.e., peak ripple power during replay epoch). For all analyses, summary effects are reported as mean ± 1 standard error of the mean (SEM), and two-tailed p < 0.05 is deemed significant, unless otherwise stated.

Statistical analysis was performed using MATLAB (Mathworks) 2019a. MEG pre-processing, time-frequency analysis and source reconstruction was performed using MATLAB in conjunction with functions from the Statistical Parametric Mapping 12 (SPM12, https://www.fil.ion.ucl.ac.uk/spm/software/spm12/) toolbox, FieldTrip (https://www.fieldtriptoolbox.org/), the OHBA Software Library (OSL, including OAT, https://ohba-analysis.github.io/osl-docs/) and FMRIB Software Library (FSL, https://fsl.fmrib.ox.ac.uk/fsl/fslwiki/).

## References

[bib1] Adams R.A., Bush D., Zheng F., Meyer S.S., Kaplan R., Orfanos S., Marques T.R., Howes O.D., Burgess N. (2020). Impaired theta phase coupling underlies frontotemporal dysconnectivity in schizophrenia. Brain.

[bib2] Altimus C., Harrold J., Jaaro-Peled H., Sawa A., Foster D.J. (2015). Disordered ripples are a common feature of genetically distinct mouse models relevant to schizophrenia. Mol. Neuropsychiatry.

[bib3] American Psychiatric Association (2013). Diagnostic and Statistical Manual of Mental Disorders.

[bib4] Barron H.C., Dolan R.J., Behrens T.E.J. (2013). Online evaluation of novel choices by simultaneous representation of multiple memories. Nat. Neurosci..

[bib5] Barron H.C., Reeve H.M., Koolschijn R.S., Perestenko P.V., Shpektor A., Nili H., Rothaermel R., Campo-Urriza N., O’Reilly J.X., Bannerman D.M. (2020). Neuronal Computation Underlying Inferential Reasoning in Humans and Mice. Cell.

[bib6] Behrens T.E.J., Muller T.H., Whittington J.C.R., Mark S., Baram A.B., Stachenfeld K.L., Kurth-Nelson Z. (2018). What Is a Cognitive Map? Organizing Knowledge for Flexible Behavior. Neuron.

[bib7] Bellmund J.L.S., Gärdenfors P., Moser E.I., Doeller C.F. (2018). Navigating cognition: Spatial codes for human thinking. Science.

[bib8] Bergel A., Tiran E., Deffieux T., Demené C., Tanter M., Cohen I. (2020). Adaptive modulation of brain hemodynamics across stereotyped running episodes. Nat. Commun..

[bib9] Bleuler E. (1911). Dementia Praecox or The Group of Schizophrenias (Dementia Praecox oder Gruppe der Schizophrenien).

[bib10] Brugger S.P., Howes O.D. (2017). Heterogeneity and Homogeneity of Regional Brain Structure in Schizophrenia: A Meta-analysis. JAMA Psychiatry.

[bib11] Buckner R.L. (2010). The role of the hippocampus in prediction and imagination. Annu. Rev. Psychol..

[bib12] Buzsáki G. (2015). Hippocampal sharp wave-ripple: A cognitive biomarker for episodic memory and planning. Hippocampus.

[bib13] Clancy M.J., Clarke M.C., Connor D.J., Cannon M., Cotter D.R. (2014). The prevalence of psychosis in epilepsy; a systematic review and meta-analysis. BMC Psychiatry.

[bib14] Constantinescu A.O., O’Reilly J.X., Behrens T.E.J. (2016). Organizing conceptual knowledge in humans with a gridlike code. Science.

[bib15] Craik K. (1943). The Nature of Explanation.

[bib16] Deuker L., Bellmund J.L., Navarro Schröder T., Doeller C.F. (2016). An event map of memory space in the hippocampus. eLife.

[bib17] Diba K., Buzsáki G. (2007). Forward and reverse hippocampal place-cell sequences during ripples. Nat. Neurosci..

[bib18] Dice L.R. (1945). Measures of the Amount of Ecologic Association Between Species. Ecology.

[bib19] Diedrichsen J., Kriegeskorte N. (2017). Representational models: A common framework for understanding encoding, pattern-component, and representational-similarity analysis. PLoS Comput. Biol..

[bib20] Dolan R.J., Dayan P. (2013). Goals and habits in the brain. Neuron.

[bib21] Dragoi G., Tonegawa S. (2013). Development of schemas revealed by prior experience and NMDA receptor knock-out. eLife.

[bib22] Dupret D., O’Neill J., Pleydell-Bouverie B., Csicsvari J. (2010). The reorganization and reactivation of hippocampal maps predict spatial memory performance. Nat. Neurosci..

[bib23] Dusek J.A., Eichenbaum H. (1997). The hippocampus and memory for orderly stimulus relations. Proc. Natl. Acad. Sci. USA.

[bib24] Eldar E., Bae G.J., Kurth-Nelson Z., Dayan P., Dolan R.J. (2018). Magnetoencephalography decoding reveals structural differences within integrative decision processes. Nat. Hum. Behav..

[bib25] Fernández-Ruiz A., Oliva A., de Oliveira E.F., Rocha-Almeida F., Tingley D., Buzsáki G. (2019). Long-duration hippocampal sharp wave ripples improve memory. Science.

[bib26] First M.B., Spitzer R.L., Gibbon M., Williams J.B.W. (1995). Structured Clinical Interview for DSM-IV Axis I disorders— Patient Edition, Version 2.

[bib27] Foster D.J. (2017). Replay Comes of Age. Annu. Rev. Neurosci..

[bib28] Foster D.J., Wilson M.A. (2006). Reverse replay of behavioural sequences in hippocampal place cells during the awake state. Nature.

[bib29] Fyhn M., Molden S., Witter M.P., Moser E.I., Moser M.B. (2004). Spatial representation in the entorhinal cortex. Science.

[bib30] Garvert M.M., Dolan R.J., Behrens T.E.J. (2017). A map of abstract relational knowledge in the human hippocampal-entorhinal cortex. eLife.

[bib31] Gelinas J.N., Khodagholy D., Thesen T., Devinsky O., Buzsáki G. (2016). Interictal epileptiform discharges induce hippocampal-cortical coupling in temporal lobe epilepsy. Nat. Med..

[bib32] Genzel L., Dragoi G., Frank L., Ganguly K., de la Prida L., Pfeiffer B., Genzel L. (2020). A consensus statement: defining terms for reactivation analysis. Philos. Trans. R. Soc. B Biol. Sci..

[bib33] Gillespie A.K., Astudillo Maya D.A., Denovellis E.L., Liu D.F., Kastner D.B., Coulter M.E., Roumis D.K., Eden U.T., Frank L.M. (2021). Hippocampal replay reflects specific past experiences rather than a plan for subsequent choice. bioRxiv.

[bib34] Grace A.A. (2016). Dysregulation of the dopamine system in the pathophysiology of schizophrenia and depression. Nat. Rev. Neurosci..

[bib35] Gupta A.S., van der Meer M.A.A., Touretzky D.S., Redish A.D. (2010). Hippocampal replay is not a simple function of experience. Neuron.

[bib36] Hafting T., Fyhn M., Molden S., Moser M.B., Moser E.I. (2005). Microstructure of a spatial map in the entorhinal cortex. Nature.

[bib37] Higgins C., Liu Y., Vidaurre D., Kurth-Nelson Z., Dolan R., Behrens T., Woolrich M. (2021). Replay bursts in humans coincide with activation of the default mode and parietal alpha networks. Neuron.

[bib38] Ho B.-C., Andreasen N.C., Ziebell S., Pierson R., Magnotta V. (2011). Long-term antipsychotic treatment and brain volumes: a longitudinal study of first-episode schizophrenia. Arch. Gen. Psychiatry.

[bib39] Howes O.D., Kapur S. (2009). The dopamine hypothesis of schizophrenia: version III--the final common pathway. Schizophr. Bull..

[bib40] Hulshoff Pol H.E., Kahn R.S. (2008). What happens after the first episode? A review of progressive brain changes in chronically ill patients with schizophrenia. Schizophr. Bull..

[bib41] Ji D., Wilson M.A. (2007). Coordinated memory replay in the visual cortex and hippocampus during sleep. Nat. Neurosci..

[bib42] Joo H.R., Frank L.M. (2018). The hippocampal sharp wave-ripple in memory retrieval for immediate use and consolidation. Nat. Rev. Neurosci..

[bib43] Kaplan R., Adhikari M.H., Hindriks R., Mantini D., Murayama Y., Logothetis N.K., Deco G. (2016). Hippocampal Sharp-Wave Ripples Influence Selective Activation of the Default Mode Network. Curr. Biol..

[bib44] Kay S.R., Fiszbein A., Opler L.A. (1987). The positive and negative syndrome scale (PANSS) for schizophrenia. Schizophr. Bull..

[bib45] Krystal J.H., Anticevic A., Yang G.J., Dragoi G., Driesen N.R., Wang X.J., Murray J.D. (2017). Impaired Tuning of Neural Ensembles and the Pathophysiology of Schizophrenia: A Translational and Computational Neuroscience Perspective. Biol. Psychiatry.

[bib46] Kurth-Nelson Z., Economides M., Dolan R.J., Dayan P. (2016). Fast Sequences of Non-spatial State Representations in Humans. Neuron.

[bib47] Lee A.K., Wilson M.A. (2002). Memory of sequential experience in the hippocampus during slow wave sleep. Neuron.

[bib48] Lewis P.A., Knoblich G., Poe G. (2018). How Memory Replay in Sleep Boosts Creative Problem-Solving. Trends Cogn. Sci..

[bib49] Liu Y., Dolan R.J., Kurth-Nelson Z., Behrens T.E.J. (2019). Human Replay Spontaneously Reorganizes Experience. Cell.

[bib50] Liu Y., Dolan R.J., Higgins C., Penagos H., Woolrich M.W., Ólafsdóttir H.F., Barry C., Kurth-Nelson Z., Behrens T.E. (2021). Temporally delayed linear modelling (TDLM) measures replay in both animals and humans. eLife.

[bib51] Liu Y., Mattar M., Behrens T.E., Daw N.D., Dolan R.J. (2021). Experience replay is associated with efficient nonlocal learning. Science.

[bib52] Luyckx F., Nili H., Spitzer B., Summerfield C. (2019). Neural structure mapping in human probabilistic reward learning. eLife.

[bib53] Maia T.V., Frank M.J. (2017). An Integrative Perspective on the Role of Dopamine in Schizophrenia. Biol. Psychiatry.

[bib54] McCutcheon R.A., Krystal J.H., Howes O.D. (2020). Dopamine and glutamate in schizophrenia: biology, symptoms and treatment. World Psychiatry.

[bib55] McCutcheon R.A., Reis Marques T., Howes O.D. (2020). Schizophrenia-An Overview. JAMA Psychiatry.

[bib56] McHugo M., Talati P., Armstrong K., Vandekar S.N., Blackford J.U., Woodward N.D., Heckers S. (2019). Hyperactivity and Reduced Activation of Anterior Hippocampus in Early Psychosis. Am. J. Psychiatry.

[bib57] McKenna P.J. (2007). Schizophrenia and Related Syndromes.

[bib58] McNamara C.G., Tejero-Cantero Á., Trouche S., Campo-Urriza N., Dupret D. (2014). Dopaminergic neurons promote hippocampal reactivation and spatial memory persistence. Nat. Neurosci..

[bib59] Morris R.W., Cyrzon C., Green M.J., Le Pelley M.E., Balleine B.W. (2018). Impairments in action-outcome learning in schizophrenia. Transl. Psychiatry.

[bib60] Nádasdy Z., Hirase H., Czurkó A., Csicsvari J., Buzsáki G. (1999). Replay and time compression of recurring spike sequences in the hippocampus. J. Neurosci..

[bib61] O’Keefe J., Dostrovsky J. (1971). The hippocampus as a spatial map. Preliminary evidence from unit activity in the freely-moving rat. Brain Res..

[bib62] O’Keefe J., Nadel L. (1978). The hippocampus as a cognitive map.

[bib63] Ólafsdóttir H.F., Barry C., Saleem A.B., Hassabis D., Spiers H.J. (2015). Hippocampal place cells construct reward related sequences through unexplored space. eLife.

[bib64] Radua J., Ramella-Cravaro V., Ioannidis J.P.A., Reichenberg A., Phiphopthatsanee N., Amir T., Yenn Thoo H., Oliver D., Davies C., Morgan C. (2018). What causes psychosis? An umbrella review of risk and protective factors. World Psychiatry.

[bib65] Roux L., Hu B., Eichler R., Stark E., Buzsáki G. (2017). Sharp wave ripples during learning stabilize the hippocampal spatial map. Nat. Neurosci..

[bib66] Schapiro A.C., McDevitt E.A., Rogers T.T., Mednick S.C., Norman K.A. (2018). Human hippocampal replay during rest prioritizes weakly learned information and predicts memory performance. Nat. Commun..

[bib67] Schmack K., Bosc M., Ott T., Sturgill J., Kepecs A. (2021). Striatal dopamine mediates hallucination-like perception in mice. Science.

[bib68] Schobel S.A., Chaudhury N.H., Khan U.A., Paniagua B., Styner M.A., Asllani I., Inbar B.P., Corcoran C.M., Lieberman J.A., Moore H., Small S.A. (2013). Imaging patients with psychosis and a mouse model establishes a spreading pattern of hippocampal dysfunction and implicates glutamate as a driver. Neuron.

[bib69] Schuck N.W., Niv Y. (2019). Sequential replay of non-spatial task states in the human hippocampus. Science.

[bib70] Silva D., Feng T., Foster D.J. (2015). Trajectory events across hippocampal place cells require previous experience. Nat. Neurosci..

[bib71] Sugden A.U., Zaremba J.D., Sugden L.A., McGuire K.L., Lutas A., Ramesh R.N., Alturkistani O., Lensjø K.K., Burgess C.R., Andermann M.L. (2020). Cortical reactivations of recent sensory experiences predict bidirectional network changes during learning. Nat. Neurosci..

[bib72] Suh J., Foster D.J., Davoudi H., Wilson M.A., Tonegawa S. (2013). Impaired hippocampal ripple-associated replay in a mouse model of schizophrenia. Neuron.

[bib73] Sun C., Yang W., Martin J., Tonegawa S. (2020). Hippocampal neurons represent events as transferable units of experience. Nat. Neurosci..

[bib74] Titiz A.S., Mahoney J.M., Testorf M.E., Holmes G.L., Scott R.C. (2014). Cognitive impairment in temporal lobe epilepsy: role of online and offline processing of single cell information. Hippocampus.

[bib75] Titone D., Ditman T., Holzman P.S., Eichenbaum H., Levy D.L. (2004). Transitive inference in schizophrenia: impairments in relational memory organization. Schizophr. Res..

[bib76] Tolman E.C. (1948). Cognitive maps in rats and men. Psychol. Rev..

[bib77] Torres U.S., Duran F.L.S., Schaufelberger M.S., Crippa J.A.S., Louzã M.R., Sallet P.C., Kanegusuku C.Y.O., Elkis H., Gattaz W.F., Bassitt D.P. (2016). Patterns of regional gray matter loss at different stages of schizophrenia: A multisite, cross-sectional VBM study in first-episode and chronic illness. Neuroimage Clin..

[bib78] van de Ven G.M., Trouche S., McNamara C.G., Allen K., Dupret D. (2016). Hippocampal Offline Reactivation Consolidates Recently Formed Cell Assembly Patterns during Sharp Wave-Ripples. Neuron.

[bib79] Van Veen B.D., van Drongelen W., Yuchtman M., Suzuki A. (1997). Localization of brain electrical activity via linearly constrained minimum variance spatial filtering. IEEE Trans. Biomed. Eng..

[bib80] Wechsler D. (2001). Wechsler test of adult reading: WTAR.

[bib81] Whitfield-Gabrieli S., Thermenos H.W., Milanovic S., Tsuang M.T., Faraone S.V., McCarley R.W., Shenton M.E., Green A.I., Nieto-Castanon A., LaViolette P. (2009). Hyperactivity and hyperconnectivity of the default network in schizophrenia and in first-degree relatives of persons with schizophrenia. Proc. Natl. Acad. Sci. USA.

[bib82] Whittington J.C.R., Muller T.H., Mark S., Chen G., Barry C., Burgess N., Behrens T.E.J. (2020). The Tolman-Eichenbaum Machine: Unifying Space and Relational Memory through Generalization in the Hippocampal Formation. Cell.

[bib83] Williams J.B.W., Kobak K.A. (2008). Development and reliability of a structured interview guide for the Montgomery Asberg Depression Rating Scale (SIGMA). Br. J. Psychiatry.

[bib84] Wilson M.A., McNaughton B.L. (1993). Dynamics of the hippocampal ensemble code for space. Science.

[bib85] Wilson M.A., McNaughton B.L. (1994). Reactivation of Hippocampal Ensemble Memories During Sleep. Science.

[bib86] Wimmer G.E., Liu Y., Vehar N., Behrens T.E.J., Dolan R.J. (2020). Episodic memory retrieval success is associated with rapid replay of episode content. Nat. Neurosci..

[bib87] Zaremba J.D., Diamantopoulou A., Danielson N.B., Grosmark A.D., Kaifosh P.W., Bowler J.C., Liao Z., Sparks F.T., Gogos J.A., Losonczy A. (2017). Impaired hippocampal place cell dynamics in a mouse model of the 22q11.2 deletion. Nat. Neurosci..

[bib88] Zeidman P., Maguire E.A. (2016). Anterior hippocampus: the anatomy of perception, imagination and episodic memory. Nat. Rev. Neurosci..

