# Supplementary material for: Impaired neural replay of inferred relationships in schizophrenia
Source: Cell. 2021 Aug 5;184(16):4315–4328.e17. doi: 10.1016/j.cell.2021.06.012 (PMC8357425; doi:10.1016/j.cell.2021.06.012)
Supplement: Document S1. Tables S1–S3 [file mmc1.pdf]

Cell, Volume *184*

Supplemental information

Impaired neural replay of inferred relationships in schizophrenia

Matthew M. Nour, Yunzhe Liu, Atheeshaan Arumuham, Zeb Kurth-Nelson, and Raymond J. Dolan

Variable	Healthy volunteers	Patients	Group comparison[#]
**Demographic**			
Sample size	27	28	
Gender	6 F, 21 M	6 F, 22 M	x^2 = 0.005 (P = 0.94)
Age (mean, SD)	27.3 (5.94)	28.2 (5.26)	t = -0.61 (P = 0.54)
Years in education (mean, SD)	18.2 (3.5)	17.0 (3.2)	t = 1.31 (P = 0.20)
Employment status [F/P/U]*	7 / 6 / 14	10 / 3 / 15	x^2 = 1.55 (P = 0.46)
Handedness	23R, 4L	26R, 2L	x^2 = 0.83 (P = 0.36)
Ethnicity [W / BAME / Other][†]	10 / 12 / 5	10 / 15 / 3	x^2 = 0.82 (P = 0.67)
Alcohol units week^{-1} (mean, SD)	3.63 (5.58)	3.18 (6.00)	t = 0.29 (P = 0.77)
Current recreational cannabis (not within 1 week)	8	11	x^2 = 0.57 (P = 0.45)
Current smoker (not within 6 hours)	5	13	x^2 = 4.86 (P = 0.03)
**Cognitive**			
IQ (SD)	103.9 (5.6)	103.9 (8.43)	t = -0.002 (P = 1.00)
Digit span forward (mean, SD)	6.35 (0.95)	6.16 (1.18)	t = 0.66 (P = 0. 52)
Digit span backward (mean, SD)	4.15 (0.99)	3.73 (1.16)	t = 1.43 (P = 0.16)
**Psychiatric symptoms and signs**			
Depressive symptoms[‡] (mean, SD)	0.89 (2.53)	8.71 (5.58)	t = -6.66 (P = $1.6*10^{-8}$)
Positive psychotic symptoms[§] (mean, SD)	7.11 (0.32)	14.4 (6.21)	t = -6.08 (P = $1.3*10^{-7}$)
Negative psychotic symptoms[§] (mean, SD)	7.07 (0.27)	14.4 (6.56)	t = -5.79 (P = $3.8*10^{-7}$)
General psychopathology[§] (mean, SD)	16.4 (1.01)	25.4 (7.08)	t = -6.51 (P = $2.8*10^{-8}$)
General assessment of functioning[‖] (mean, SD)	98.3 (5.2)	69.4 (14.2)	t = 9.95 (P = $1.0*10^{-13}$)
**Clinical Details**			
Number taking D2/3R antagonist medication	-	15[¶]	-
Months since first psychotic episode (median, IQR)	-	48 (30)	-
Number acute psychotic episodes (median, IQR)	-	3 (2)	-
Number inpatient admissions (median, IQR)	-	1 (3)	-

Table S1. Participant demographic, cognitive and clinical information. Related to STAR*Methods.

[*] F = fulltime employment, P = part-time employment, U = unemployed (including student).
[†] W = White. BAME = Black, Asian, and Minority Ethnic. Other includes multiple ethnic groups.
[‡] Montgomery Åsberg Depression Rating Scale (MADRS), floor = 0.
[§] Positive and Negative Syndrome Scale (PANSS) scale, floor = 7(positive), 7(negative), 16(general).
[‖] General Assessment of Functioning (GAF) scored from 0 – 100.
[¶] D2/3 antagonist medication per medicated patient: (1) olanzapine 15 mg day^{-1}, (2) olanzapine 10 mg day^{-1}, (3) lurasidone 18.5 mg day^{-1}, (4) aripiprazole 10mg day^{-1}, (5) lurasidone 37 mg day^{-1}, (6) risperidone 3 mg day^{-1}, (7) aripiprazole 400mg month^{-1} (depot), (8) risperidone 0.5 mg day^{-1}, (9) aripiprazole 5 mg day^{-1}, (10) olanzapine 7.5 mg day^{-1}, (11) olanzapine 10 mg day^{-1}, (12) amisulpride 400 mg day^{-1} & aripiprazole 5 mg day^{-1}, (13) paliperidone 50 mg month^{-1} (depot), (14) paliperidone 175 mg 3-month^{-1} (depot), (15) paliperidone 50 mg month^{-1} (depot).
[#]Group comparisons: unpaired t-test for continuous variables (two-tailed), Chi squared test for categorical variables (two-tailed).
SD: standard deviation. IQR: inter-quartile range.

Regression model (predictor variables)	beta	S.E	T stat (df)	P value
**Position representation**				
a. Position ~ group + peak ripple power (PRE) + interaction				
Group*	0.017	0.012	1.45, (49)	0.16
Peak ripple power[†] (PRE[§])	0.008	0.009	0.92 (49)	0.36
Interaction	-0.022	0.018	-1.24 (49)	0.22
b. Position ~ group + sequenceness (POST) + interaction				
Group	0.017	0.013	1.30 (50)	0.20
Sequenceness[‡] (POST[§])	0.001	0.003	0.38 (50)	0.71
Interaction	-0.0003	0.006	-0.051 (50)	0.96
**Within-sequence confusion representation**				
a. Within-sequence confusion ~ group + sequence learning efficiency + interaction				
Group	-0.016	0.009	-1.64 (51)	0.11
Sequence learning efficiency	-0.038	0.037	-1.03 (51)	0.31
Interaction	0.020	0.073	0.26 (51)	0.79
b. Within-sequence confusion ~ group + peak ripple power (POST) + interaction				
Group	-0.022	0.009	-2.32 (49)	0.03
Peak ripple power (POST)	-0.005	0.007	-0.69 (49)	0.49
Interaction	-0.015	0.013	-1.11 (49)	0.27
c. Within-sequence confusion ~ group + sequenceness (POST) + interaction				
Group	-0.019	0.011	-1.87 (50)	0.07
Sequenceness (POST)	0.0005	0.002	0.20 (50)	0.84
Interaction	-0.002	0.005	-0.35 (50)	0.73
**Across-sequence confusion representation**				
a. Across-sequence confusion ~ group + sequence learning efficiency + interaction				
Group	0.005	0.008	0.66 (51)	0.51
Sequence learning efficiency	0.003	0.032	0.79 (51)	0.94
Interaction	0.077	0.063	1.22 (51)	0.22
b. Across-sequence confusion ~ group + peak ripple power (POST) + interaction				
Group	$5.56*10^{-5}$	0.008	0.007 (49)	0.99
Peak ripple power (POST)	-0.005	0.006	-0.83 (49)	0.41
Interaction	0.002	0.012	0.18 (49)	0.86
c. Across-sequence confusion ~ group + sequenceness (POST) + interaction				
Group	0.002	0.008	0.22 (50)	0.83
Sequenceness (POST)	-0.002	0.002	-1.10 (50)	0.28
Interaction	0.004	0.004	0.89 (50)	0.38

Table S2. Control analyses for prediction of structural representation post-learning. Related to Figures 7 and S5.
**Group* is effects coded (patients = -0.5, controls = +0.5).
[†]*Peak ripple power* is the peak power increase in the ripple band, within the presumptive replay epoch (0 – 50 ms ± 10 ms), mean centered.
[§]*PRE & POST* refer to pre- and post-learning rest sessions.
[‡]*Sequenceness* is the mean effect at 40 – 50 ms (peak in the combined sample).

Regression models grouped by dependent variable (representation effect at predictor-specific peak time point, derived from combined sample, see **Figures 7 & S5**). Three predictor variables per model: two main effects and their interaction.
S.E. = standard error of beta estimate.
df = degrees of freedom associated with t-statistic on the coefficient.

Clinical variable	Correlation* with Sequence Learning Efficiency	Correlation with Sequenceness (POST learning, 40-50 ms lag)
**Symptom scores**		
Positive psychotic symptoms (PANSS†)	rho(26) = -0.21, P = 0.27	rho(25) = 0.11, P = 0.60
Negative psychotic symptoms (PANSS)	rho(26) = 0.05, P = 0.79	rho(25) = -0.04, P = 0.83
General psychopathology (PANSS)	rho(26) = -0.23, P = 0.24	rho(25) = -0.17, P = 0.40
Depressive symptoms (MADRS‡)	rho(26) = -0.12, P = 0.54	rho(25) = -0.19, P = 0.33
**Illness chronicity**		
Months since first psychotic episode	rho(26) = 0.16, P = 0.41	rho(25) = 0.23, P = 0.24
Age at symptom onset	rho(26) = 0.11, P = 0.57	rho(25) = 0.14, P = 0.50
Age at MEG	rho(26) = 0.05, P = 0.81	rho(25) = 0.15, P = 0.47

Table S3. Correlation between clinical variables and primary behavioral and neural measures in patients. Related to Figures 2 & 4.

[*] Spearman’s rank correlation coefficient.

[†] Positive and Negative Syndrome Scale (PANSS) scale.

[‡] Montgomery Åsberg Depression Rating Scale (MADRS).
